# Designing a Robust MEA-Based Post-Combustion Carbon Capture Process with Capture Rate Guarantees

**DOI:** 10.1021/acs.iecr.6c00102

**Published:** 2026-06-16

**Authors:** Jason A. F. Sherman, Anca G. Ostace, Douglas A. Allan, Chrysanthos E. Gounaris

**Affiliations:** † Department of Chemical Engineering, 6612 Carnegie Mellon University, Pittsburgh, Pennsylvania 15213, United States; ‡ 17213 National Energy Technology Laboratory, Pittsburgh, Pennsylvania 15236, United States; ¶ National Energy Technology Laboratory Support Contractor, Pittsburgh, Pennsylvania 15236, United States

## Abstract

The development and widespread commercial deployment of carbon capture and storage technologies will be instrumental in expanding affordable energy production and increasing the availability of CO_2_ as a feedstock for several industrial applications. This development can be accelerated by applying computational model-based process optimization methodologies that explicitly account for the impact of parametric uncertainties to obtain solutions that exhibit minimal technical risk. Robust optimization (RO) is one such prominent methodology. In this work, we present a successful application of the nonlinear two-stage RO solver PyROS to a detailed rate-based, equation-oriented model for the economical design and operation of a monoethanolamine scrubbing process for postcombustion carbon capture under uncertainty in the thermodynamic property submodel parameters. Our application enables us to successfully obtain risk-averse model solutions for CO_2_ capture targets ranging from 90% to over 99%, with solutions for capture targets of up to 98% only marginally more expensive than their nominally optimal counterparts. Thus, our results demonstrate that employing RO and the PyROS solver can help us obtain risk-averse carbon capture process designs without inherently unnecessary cost burdens that are often associated with ad hoc overdesign approaches.

## Graphical abstract

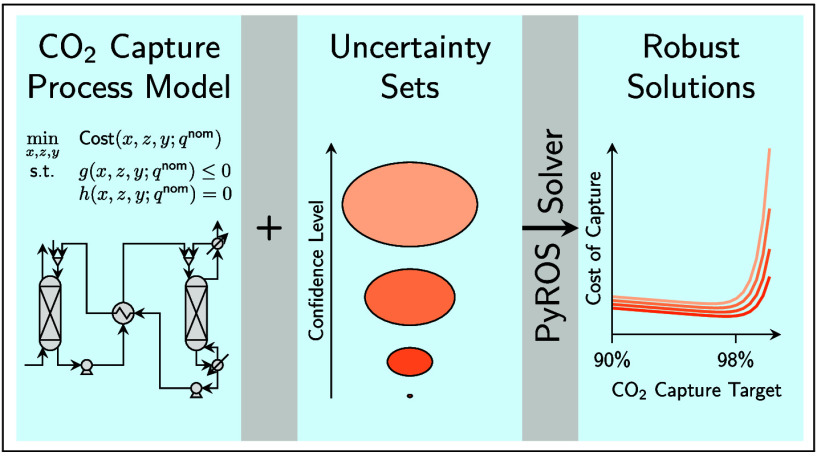

## 1 Introduction

Carbon capture and storage (CCS) technologies, when developed and deployed commercially to remove CO_2_ from conventional fossil energy systems, are expected to help expand affordable energy production and the availability of CO_2_ as a feedstock in several industrial applications, such as enhanced oil recovery and synthetic fuel production.^1–3^ Historically, the scale-up and deployment of a new technology in the energy sector requires 20–30 years of effort.^4^ The incorporation of advanced model-based approaches into the technology development process has been shown to dramatically reduce the time and monetary expenditure of scale-up and deployment in other applications in the energy sector.^4,5^ Therefore, computational modeling approaches can potentially reduce the resource investments required to develop and deploy new and existing CCS technologies while enabling the quantification and reduction of technical risk.

Modeling of amine scrubbing processes for postcombustion CO_2_ capture can serve as a benchmark for the development and deployment of new CCS technologies. While commercial-scale amine scrubbing processes have been used to treat gaseous mixtures since 1930,^2,6,7^ using 30 wt % aqueous monoethanolamine (MEA) as the solvent in commercial-scale amine scrubbing processes has been widespread since 1970.^8^ Presently, there are several commercial-scale postcombustion CO_2_ amine scrubbing projects in operation worldwide.^1–3,9^ As a consequence, the scientific literature contains a large body of empirical process and property data on amine scrubbing,^8^ and open access to these data facilitates the formulation and validation of rigorous multiscale process models. This is very important, inasmuch as workflows based on these models are transferable to the subsequent development of other CCS technologies.

Computational modeling approaches for the design and operation of postcombustion CO_2_ amine scrubbing processes have been extensively studied in the scientific literature over the past few decades. A number of published works are comprehensively reviewed in Chatziasteriou et al.^10^ and predominantly involve the development and use of steady-state models in which the amine solvent is aqueous MEA. We summarize a select few such works here. In Mores et al.^11^ lies the development of a steady-state model for economically optimizing the design and operation of an MEA scrubbing process coupled with a natural gas combined cycle (NGCC) power plant for CO_2_ capture rates ranging from 70% to 95%. A systematic procedure for steady-state modeling, design, and techno-economic analysis of an MEA scrubbing process using the Aspen Plus process simulator was demonstrated in Madeddu et al.^12^ More recently, Akula et al.^13^ developed a rate-based, equation-oriented model for optimizing the operating costs of MEA scrubbing processes fitted to pulverized coal and NGCC power plants under part-load and variable CO_2_ capture operations, subject to CO_2_ capture rates of 75% and 90%. Finally, the work Isenberg et al.^14^ presented a rate-based, equation-oriented model for optimizing the design and operation of an MEA scrubbing process, subject to a CO_2_ capture target of 85%.

Typical commercial-scale deployments of amine scrubbing processes currently in operation have aimed for 85–90% capture.^2,3^ However, recent works have postulated that the technical and economic barriers to achieving even higher CO_2_ capture rates are surmountable.^15–18^ “Ultrahigh” capture, a term often used to describe CO_2_ capture rates of 99% or higher, can be achieved through various process alterations, such as the installation of larger process units or usage of a different amine solvent,^15,16,19^ as has been demonstrated also from a modeling perspective.^20,21^

Quantifying and explicitly accounting for parametric uncertainty in the solution of process models for amine scrubbing is crucial for obtaining risk-averse designs. As concretely shown in prior rigorous uncertainty quantification and sensitivity analysis studies,^8,22–24^ fluctuations in the transport and thermodynamic property parameters, for example, may significantly alter key outputs of rigorous MEA scrubbing process models, such as the CO_2_ capture rate and lean solvent CO_2_ loading. Thus, designs that are optimal or feasible for only a nominal realization of the uncertain model parameters may become mathematically infeasible or suboptimal subject to off-nominal uncertain parameter realizations. Implementing such designs in practice may result in significant cost incurrences or violations of operational performance and safety requirements when the uncertain parameters deviate from their nominal values.^8,14,22–24^ More broadly, the importance of quantifying, accounting for, and reporting uncertainty in techno-economic analyses of CCS process models is greatly accentuated by the novelty of many CCS technologies.^25^

Robust optimization (RO) is a suitable approach for obtaining economical, risk-averse solutions to amine scrubbing process models subject to parametric uncertainty. Popularized by the work of Ben-Tal and Nemirovski^26^ for the solution of uncertain linear programs, RO is a modeling approach that seeks, for a given model, the best solution that remains feasible, subject to every realization of the uncertain parameters within a predefined set. RO frameworks and solution methodologies for general nonlinear programs have been studied since the 2000s; for a comprehensive survey, the reader is referred to Leyffer et al.^27^ A method based on linearization with respect to the uncertain parameters about their nominal values was proposed by Zhang;^28^ this method was later refined and extended by Yuan et al.^29^ More recently, Isenberg et al.^14^ presented a generalization of the cutting-set approach of Mutapcic and Boyd^30^ to make it suitable for application on nonconvex models often used for process system design. The above nonlinear RO approaches are applicable to models with uncertain nonlinear constraints, including equalities, and with operational decision variables that can be adjusted to uncertain parameters. Indeed, many amine scrubbing process models satisfy these characteristics.

Nonlinear RO has been successfully used to obtain risk-averse solutions to computational process design and operation models in previous works. Hale and Zhang^31^ applied the method of Zhang^28^ to the design of a heat exchanger network and a reactor–separator system. The refined linearization approach of Yuan et al.^29^ was applied to the design problems of Hale and Zhang^31^ and a reactor–heat exchanger system. In Kammammettu and Li,^32^ the algorithm of Yuan et al.^29^ was further improved and applied to the design and operation of water treatment networks. In Isenberg et al.,^14^ the cutting-set algorithm presented was applied to three robust process design models, including a complex model of an MEA scrubbing process for CO_2_ capture under uncertainty in the thermodynamic property model parameters. The algorithm of Isenberg et al.^14^ later formed the basis for the open-source two-stage RO solver PyROS,^33^ which has been applied to the solution of a detailed MEA absorption column model under uncertainty in the thermodynamic property submodel parameters^34^ and to an ultrapure water production plant model under aleatoric uncertainty.^35^

Applications of nonlinear RO to the design of amine scrubbing systems presented in the existing literature appear to be limited in number and scope. The works of Cerrillo-Briones and Ricardez-Sandoval^36^ and Sherman et al.^34^ present studies of a standalone absorption column, subject to CO_2_ capture targets ranging from 89% to 95%, under uncertainty in either thermodynamic equilibrium or flue gas property parameters. The case study of Isenberg et al.^14^ is, to our knowledge, the only published article demonstrating a successful application of RO to the model-based design of a full amine scrubbing flowsheet; however, that case study was performed subject to a modest CO_2_ capture target of 85% and uncertainty in only two thermodynamic equilibrium parameters. In light of the preceding exposition, we find interest in the application of RO to the risk-averse design of an amine scrubbing process subject to a broad range of high, including ultrahigh, CO_2_ capture targets and immunized against a broader set of sources of uncertainty. However, using an RO solver to reliably obtain risk-averse solutions to a detailed amine scrubbing process model subject to such a range of CO_2_ capture targets and uncertainty quantifications requires that the model formulation and implementation first be made numerically robust. Achieving this numerical robustness requires a significant undertaking that involves the application of modeling techniques similar to those presented by Allan et al.^37^

In this work, we present a computational study of our application of the robust optimizer PyROS^33^ to the solution of a novel high-fidelity optimization model for the design and operation of a conventional MEA scrubbing process, fitted to a net 727 MWe NGCC power plant under uncertainty in the thermodynamic property submodel parameters. The process model is obtained by modifying that of Akula et al.^13^ for the purpose of enhanced numerical robustness, facilitating the automatic solution of the model using PyROS. The associated costs measuring the economic feasibility of the process are estimated through key metrics related to the total annualized cost and levelized cost of capture. Further, to estimate the additional expenditure of fitting the scrubbing process to the NGCC plant, the cost of capture and levelized cost of electricity of the coupled NGCC plant and scrubbing process are also reported, as prescribed in the National Energy Technology Laboratory (NETL) Fossil Energy Baseline Report.^38,39^ We show that PyROS enables us to obtain risk-averse designs for the scrubbing process, subject to CO_2_ capture targets ranging from 90% to 99.3%. These risk-averse designs are shown to be operationally robust and, for CO_2_ capture targets of up to 98%, only marginally more expensive than their nominally optimal counterparts.

The remainder of this article is organized as follows. In Section 2, we formally describe the process model and quantification of parametric uncertainty. In Section 3, we present a computational study of the model for a range of CO_2_ capture targets and detailed comparisons of model solutions obtained using PyROS. Some final conclusions are provided in Section 4.

## 2 Problem Statement

### 2.1 Process Description

The system of interest is described as follows. Flue gas from a 727 MWe NGCC power plant is pretreated to a pressure of 0.105 MPa, temperature of 40 °C, and composition of 4.226 mol % CO_2_, 5.48 mol % H_2_O, 77.864 mol % N_2_, and 12.43 mol % O_2_, in accordance with the specifications of the 90% CO_2_ capture NGCC plant case study of James III et al., Schmitt et al.^38,39^ To efficiently handle its large quantity, the pretreated flue gas is split evenly into four streams to be treated by four identical scrubbing process trains. At a flow rate of 9.55 × 10^5^ kg/h, each stream of pretreated flue gas enters the MEA scrubbing process of interest (Figure 1). Upon entering the process, the flue gas stream is fed to an absorption column in counter-current flow against a stream of aqueous MEA solvent, into which much of the CO_2_ is absorbed, while the cleaned gas is emitted to the atmosphere. The resulting stream of CO_2_-rich solvent is pumped through a cross heat exchanger, in which the CO_2_-rich solvent cools a stream of CO_2_-lean solvent recycled from a stripping column. Through the application of hot water vapor in the stripping column, the absorbed CO_2_ is removed from the solvent. The stripping vapor is partially condensed to isolate the CO_2_, which is finally compressed prior to transport.

**Figure 1:**
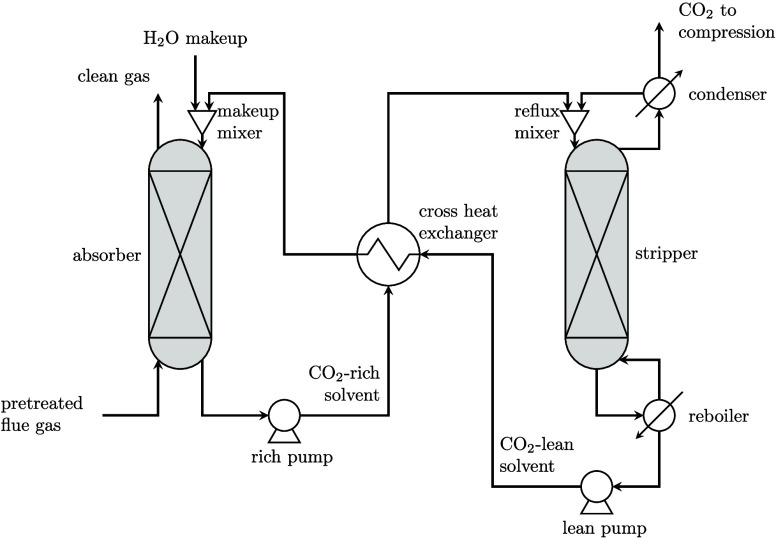
Flowsheet of a conventional amine scrubbing process for postcombustion CO_2_ capture.

### 2.2 Deterministic Optimization Model

Our process optimization model is summarized as follows: Given the flue gas conditions described previously and a CO_2_ capture target, the goal is to optimize the design and operation of the process for the minimization of the levelized cost of target capture (LCOTC). The model features technical constraints governed by the underlying physics of the process and one or more environmental constraints based on the specified CO_2_ capture target.

Mathematically, our deterministic MEA scrubbing process optimization model is a nonlinear program (NLP) of the form

1 $$\begin{array}{ccc} \underset{\begin{array}{c} \begin{array}{c} x\in X\\ z\in R^{n_{z}} \end{array}\\ y\in R^{n_{y}} \end{array}}{\min} & \mathrm{LCOTC}\left(x,z,y\right) & \left(1a\right)\\ \mathrm{s.t.} & g\left(x,z,y\right)\leq 0 & \left(1b\right)\\ & h\left(x,z,y;q^{\mathrm{nom}}\right)=0 & \left(1c\right) \end{array}$$

where:

-
$x\in R^{n_{x}}$ denotes the design, or *first-stage*, decision variables, which comprise the absorber and stripper diameters and packed heights, heat exchanger areas, maximum rich and lean solvent volumetric flow rates, maximum reboiler heat duty, maximum mass flow rate of CO_2_ capture, and maximum solvent fill.
-
$z\in R^{n_{z}}$ denotes the operational, or *second-stage* (recourse), decision variables, which comprise the MEA component recirculation rate, condenser cooling water utility flow rate, and reboiler steam utility flow rate.
-
$y\in R^{n_{y}}$ denotes the state variables, which include the solvent loadings, CO_2_ capture rate, stream temperatures, and stream pressures, among others.
-
$q^{\mathrm{nom}}\in R^{n_{q}}$ refers to the nominal realization of the model’s uncertain parameters, which are discussed further in the next subsection.
-
$X\subseteq R^{n_{x}}$ denotes a set defined by constraints imposed on *x* only, such as practice-motivated bounds on column dimensions and heat exchanger areas.
-
$\mathrm{LCOTC}:X\times R^{n_{z}}\times R^{n_{y}}\to R$ is a function defining the LCOTC of the scrubbing process.
-
$g:X\times R^{n_{z}}\times R^{n_{y}}\to R^{n_{g}}$ is a vector of the operational inequality constraint functions, which specify the operational performance requirements and consist of a lower bound constraint for the CO_2_ capture rate; lower and upper bound constraints for the flooding fraction throughout the absorber and stripper; operational capacity constraints for the reboiler duty, solvent volumetric flows, CO_2_ capture mass flow rate, and MEA recirculation rate; and non-negativity constraints for the heat exchanger utility flows and heat exchanger end temperature deltas.
-
$h:X\times R^{n_{z}}\times R^{n_{y}}\times R^{n_{q}}\to R^{n_{h}}$ is a vector of the operational equality constraint functions, which define the state variables *y*; these constraints consist of the material and energy balances, along with the equations defining the costing, transport property, thermodynamic property, and kinetic property submodels.

Note that, in the deterministic model (eq 1), the uncertain parameters appear only in the operational equality constraints (eq 1c). A design x∈X is said to be *deterministically feasible* or *nominally feasible* if there exist $z\in R^{n_{z}},y\in R^{n_{y}}$ such that (*x*, *z*, *y*) is a feasible solution to eq 1.

The model is described in more detail in the remainder of this section. In section 2.2.1, we formally describe the technical portion of the model, whereas in section 2.2.2, we formally define the LCOTC and discuss other economic aspects of the model.

#### 2.2.1 Technical Considerations

The technical portion of the MEA-based carbon capture model used in this study is obtained by modifying that of the model of Akula et al.^13^ for enhanced numerical robustness, to facilitate automated solution of a derived RO counterpart with PyROS. The absorber and stripper columns, both modeled as one-dimensional steady-state, rate-based packed columns spatially discretized along the lengths of the columns, consist of vapor and liquid molar component balances and enthalpy balances. The vapor and liquid molar component balances are given by

$$\frac{d\left(u_{V}C_{i,V}\right)}{d\zeta }=-N_{i,V}\forall i\in \{\begin{array}{cc} \left\{{\mathrm{CO}}_{2},H_{2}O,N_{2},O_{2}\right\} & \left(\mathrm{absorber}\right)\\ \left\{{\mathrm{CO}}_{2},H_{2}O\right\} & \left(\mathrm{stripper}\right) \end{array}$$ 2

and

3 $$\frac{d\left(u_{L}C_{i,L}^{a}\right)}{d\zeta }=-N_{i,L}\forall i\in \left\{{\mathrm{CO}}_{2},H_{2}O,\mathrm{MEA}\right\}$$

where *u*_V_ and *u*_L_ denote the vapor and liquid phase superficial velocities, respectively; *C*_*i*,V_ denotes the molar concentration of species *i* in the vapor phase; *C*_*i*,L_^a^ denotes the molar concentration of the apparent species *i* in the liquid phase; *N*_*i*,V_ and *N*_*i*,L_ denote the vapor and liquid phase molar fluxes of species *i*, respectively; and ζ denotes the axial coordinate in the column's spatial domain. The apparent species are related to the true ionic species present in the liquid phase through the speciation model briefly described later in this section (full details can be found in Akula et al.^13^). The steady-state enthalpy balances for the vapor and liquid phases are

$$\frac{d\left(u_{V}C_{\mathrm{tot},V}{Ĉ}_{p,V}T_{V}\right)}{d\zeta }=Q_{V}$$ 4

and

$$\frac{d\left(u_{L}C_{\mathrm{tot},L}{Ĉ}_{p,L}T_{L}\right)}{d\zeta }=Q_{L}$$ 5

where *Q*_V_ and *Q*_L_ represent the interphase heat transfer terms, which account for convective heat transfer between the phases as well as the latent heat contributions associated with condensation and evaporation of water; *C*_tot,V_ and *C*_tot,L_ represent the total molar concentrations of the vapor and liquid phases, respectively; *Ĉ*_*p*,V_ and *Ĉ*_*p*,L_ represent the vapor and liquid phase molar heat capacities, respectively; and *T*_V_ and *T*_L_ represent the vapor and liquid phase temperatures, respectively.

Both the flue gas and the stripper boilup are treated as ideal gas mixtures, while MEA is assumed to be nonvolatile and is therefore absent from the vapor phase. The interphase mass and heat transfer rates are computed algebraically using correlations for the mass transfer coefficients and interfacial area, as listed in Table 3 of Akula et al.^13^ Enhancement of CO_2_ mass transfer due to chemical reaction in the liquid film is described using an approximation of the general enhancement factor model of Gaspar and Fosbøl.^37,40^ As discussed in detail in Allan et al.,^37^ the original implicit formulation of this model exhibits a 0/0 singularity when the system approaches vapor–liquid equilibrium (i.e., when the bulk liquid-phase CO_2_ vapor pressure equals the vapor-phase CO_2_ partial pressure), which is extremely troublesome for NLP solvers and is hard to remove. Since the limit of the enhancement factor as the system approaches equilibrium is finite and possesses a closed-form expression, this equilibrium-limit approximation is adopted in place of the full implicit model. This approximation is expected to be adequate for the high-capture conditions of interest in this work, where the system operates near equilibrium pinch conditions throughout much of the column. We refer readers to Section 4 of Allan et al.^37^ for a more detailed discussion of this enhancement factor approximation strategy.

Liquid-phase speciation in the bulk is governed by the reversible bicarbonate (HCO_3_^–^) and carbamate (MEACOO^–^) formation reactions^13,23^

6 $$\mathrm{MEA}+{\mathrm{CO}}_{2}+H_{2}O\overset{K_{1}}{⇌}{\mathrm{MEAH}}^{+}+{\mathrm{HCO}}_{3}^{-}$$

and

7 $$2\mathrm{MEA}+{\mathrm{CO}}_{2}\overset{K_{2}}{⇌}{\mathrm{MEAH}}^{+}+{\mathrm{MEACOO}}^{-}$$

where *K*_1_ and *K*_2_ are the concentration-based chemical reaction equilibrium constants. Chemical equilibrium is assumed to prevail in the liquid bulk, with the true species distribution determined by the constants *K*_1_ and *K*_2_; species balances for MEA, CO_2_, and H_2_O; and an electroneutrality condition. Reaction kinetics are accounted for solely through the enhancement factor in the mass transfer rate expressions. In contrast to Akula et al.,^13^ who used the rate constants of Luo et al.,^41^ the present work adopts those of Putta et al.,^42^ which through an internal study have been observed to yield improved agreement with experimental data and better numerical robustness.

Finally, the thermodynamic property models used to describe the vapor and liquid phases of the CO_2_–MEA–H_2_O capture system and the transport properties for the rate-based model are summarized in Table 1. The vast majority of the correlations used in the capture model have been reformulated using logarithmic-form variables to improve variable scaling and aid computational tractability. For details on this process, see Section 3 of Allan et al.^37^

**Table 1:** References for Thermodynamic and Transport Properties Used to Model the CO_2_–MEA–H_2_O System^a^

thermodynamic property	reference
density	Weiland et al.^43^ and Morgan et al.^22^
heat capacity	Correlated to data from Hilliard^44^
CO_2_ Henry’s law constant	Jiru et al.^45^
equilibrium constants	Correlated to model from Morgan et al.^23^
H_2_O vapor pressure	DIPPR^46^
enthalpy of absorption	Kohl et al.^47,48^ and Akula et al.^13^

transport property	reference
viscosity	Weiland et al.^43^ and Morgan et al.^22^
pure component thermal conductivity	Reid et al.^49^
thermal conductivity mixing rule	Li^50^
CO_2_ liquid diffusivity	Ying and Eimer^51^
MEA liquid diffusivity	Snijder et al.^52^
electrolyte liquid diffusivity	Hoff et al.^53^
surface tension	Asprion^54^ and Morgan et al.^22^

a Adapted from Akula et al.^13^ Copyright © 2026 American Chemical Society.

The cross heat exchanger between the rich and lean solvent streams is modeled using a constant overall heat transfer coefficient and log mean temperature difference. The condenser and reboiler are each modeled as single equilibrium stages, using the IDAES SolventCondenser and SolventReboiler models, respectively, which incorporate component and energy balances together with vapor–liquid equilibrium and the same liquid-phase speciation as the column models. The condenser temperature difference is computed assuming a constant condensate-side temperature equal to the condensate outlet temperature. The reboiler is heated by condensing steam bled from the NGCC’s steam cycle, so the reboiler’s temperature difference is calculated as the difference between the steam condensate’s temperature and the column boilup’s temperature. The remaining mixer and pump models are from the IDAES library. Because pressure drop across units is small, it is not calculated.

#### 2.2.2 Economic Considerations

We now discuss the model’s economic aspects, which comprise (the calculations of) the LCOTC and other costing metrics. The LCOTC is calculated using

$$\mathrm{LCOTC}\left(x,z,y\right)=\frac{\mathrm{TAC}\left(x,z,y\right)}{{ṁ}_{CO_{2}}^{\mathrm{targ}}}$$ 8

in which TAC(x,z,y) denotes the total annualized cost (TAC) and *ṁ*_CO_2__^targ^ denotes the minimum required mass flow rate of CO_2_ captured from the flue gas. Note that we optimize for the levelized cost of *target* capture rather than the more traditionally used levelized cost of capture (LCOC), which is defined by

$$\mathrm{LCOC}\left(x,z,y\right)=\frac{\mathrm{TAC}\left(x,z,y\right)}{{ṁ}_{CO_{2}}}$$ 9

in which *ṁ*_CO_2__ is a state variable denoting the actual mass flow rate of CO_2_ captured from the flue gas. The reason for doing so is that, since the CO_2_ capture flow rate *ṁ*_CO_2__ is the denominator of the LCOC, optimizing for the latter may incentivize maximizing *ṁ*_CO_2__ beyond the required threshold *ṁ*_CO_2__^targ^ at the expense of TAC minimization, which is more economically desirable.

The total annualized cost is defined using the formulation of Hasan et al.,^55^ that is, using the equation

10 TAC(x,z,y)=AIC(x)+AOC(z,y)

where AIC(x) is the annualized investment cost (AIC) in $/yr, which depends on the first-stage decision variables *x*, and AOC(z,y) is the annualized operating cost (AOC) in $/yr, which depends on the consumption rates of process utilities. Further, the AIC is given by

11 AIC(x)=ϕ·TPC(x)+AMC(x)

in which TPC(x) denotes the total plant cost (TPC) in $, the quantity ϕ is a constant denoting the capital recovery factor in yr^–1^, and AMC(x) denotes the annual maintenance cost (AMC) in $/yr. The TPC accounts for the specifications of the process units comprising the flowsheet of Figure 1, along with the costs of solvent filtration and storage. As in Hasan et al.,^55^ the magnitude of the AMC is taken to be 5% of that of the TPC. Annualization of the TPC is performed under the assumption of a 25-year process lifetime and an annual interest rate of 8%; hence, we take ϕ ≈ 0.0937.

A more comprehensive economic submodel is also implemented as prescribed in the NETL Fossil Energy Baseline Report^38,39^ to systematize the estimation and communication of the costs of the NGCC plant and parallel scrubbing processes (CCS system), the combination of which we refer to as the NGCC+CCS system. We note that the NGCC plant is represented in the economic submodel only and is implemented as a surrogate model trained on simulation data obtained using the Aspen Plus model of the Case B31A NGCC steam cycle presented in the NETL Baseline Report.^38^ The extended economic submodel is implemented for simulation and economic evaluation of the NGCC+CCS system given a (feasible) solution to the deterministic model (eq 1) of the scrubbing process. As per the NETL guidelines,^38,39^ the comprehensive economic model calculates costs and the levelized cost of electricity (LCOE, in $/MWh) of a reference NGCC plant without a fitted CCS system and that of the NGCC+CCS system, which we denote by ${\mathrm{LCOE}}_{\mathrm{NGCC}}$ and ${\mathrm{LCOE}}_{\mathrm{NGCC}+\mathrm{CCS}}\left(x,z,y\right)$ , respectively. Based on the difference between the LCOE values of the two systems, we then report the cost of capture (COC) in $/metric ton using

$$\mathrm{COC}\left(x,z,y\right)=\frac{{Ẇ}_{\mathrm{NGCC}}^{\mathrm{net}}}{{ṁ}_{CO_{2}}^{\mathrm{targ}}}\cdot \left({\mathrm{LCOE}}_{\mathrm{NGCC}+\mathrm{CCS}}\left(x,z,y\right)-{\mathrm{LCOE}}_{\mathrm{NGCC}}-\mathrm{LTC}\left(x,z,y\right)\right)$$ 12

where LTC⁢(x, z, y) denotes the levelized transportation cost (LTC) in $/MWh and *Ẇ*_NGCC_^net^ denotes the net power production of the NGCC plant in MW.

The LCOE for the NGCC+CCS system comprises the levelized capital cost, levelized fixed operation and maintenance cost, and levelized variable operation and maintenance cost. The capital and project costs required to calculate the LCOE are based on the capital and operation and maintenance costs of the NGCC+CCS system, as described in the Quality Guidelines for Energy System Studies (QGESS) cost estimation methodology.^56^ The economic model for the NGCC+CCS system includes correlations to compute the total as spent capital, total overnight capital, owner’s cost (preproduction, inventory capital, land, financing), total plant cost, and bare erected cost. For details on the calculation of the capital and project costs of the NGCC+CCS plant, the reader is referred to the QGESS reports^56,57^ and NETL’s CCS capital costing methodology.^58^ In the more comprehensive economic model, the equipment considered in the capital cost calculation includes both CO_2_ capture and flue gas pretreatment equipment, in line with the NETL baseline report.^38^ The pretreatment equipment considered is the flue gas blower, direct contact cooler, and direct contact cooler pump. The capture equipment considered is the absorber, cross heat exchanger, stripper, stripper condenser, stripper reboiler, and other equipment related to solvent treatment, storage, and transport. Additional line items that are essential for plant operation, such as accessory electric plants, instrumentation and control, site improvements, buildings and structures, foundations, and interconnecting piping, are also included in the TPC.

The reference NGCC plant used in the LCOE and COC calculations is Case B31A of the NETL Fossil Energy Baseline Report.^38,39^ Case B31A represents a plant that produces a net output of 727 MWe at a net plant efficiency of 53.6%, on a higher heating value (HHV) basis. The plant’s major equipment list and detailed cost breakdown are presented in the NETL Fossil Energy Baseline Report.^38,39^ The LCOE of the reference NGCC plant was $43.3/MWh. For the study presented in this paper, the NGCC+CCS plant configuration is the same as that of Case B31A, with the distinction that the 30 wt % MEA-based CCS is added for CO_2_ capture. The CCS facility increases the auxiliary power load on the NGCC plant, thereby reducing the NGCC plant’s power output. The CCS facility’s steam requirements are satisfied by extracting steam from the intermediate-low-pressure (IP/LP) crossover of the NGCC plant.

### 2.3 Uncertainty Quantification

As previously summarized in Table 1, the amine scrubbing process model consists of submodels for several transport and thermodynamic properties. Prior studies of sources of uncertainty in these submodels showed that the predictions of the full process model are most affected by parametric uncertainty in the thermodynamic property submodels.^22–24^ In advance of the present study, we performed an analysis of the effect of uncertainty in the parameters of the solution density, viscosity, surface tension, chemical reaction equilibrium, and vapor–liquid equilibrium (VLE) submodels. As in prior publications,^22–24^ our analysis revealed that the uncertainty in the parameters of the viscosity, density, and surface tension submodels have an insignificant effect on the model predictions, compared to that of the uncertainty in the VLE and chemical reaction equilibrium submodel parameters. Thus, we exclusively consider uncertainty in the VLE and chemical reaction equilibrium parameters in the present study.

Uncertainty in the VLE submodel arises from the equations governing the solubility of CO_2_ at equilibrium. Assuming ideal behavior, the solubility of CO_2_ at equilibrium is governed by Henry’s law,

13 $$y_{{\mathrm{CO}}_{2}}P=H_{{\mathrm{CO}}_{2}}C_{{\mathrm{CO}}_{2},L}^{t}$$

where *y*_CO_2__ denotes the mole fraction of CO_2_ in the vapor phase, *P* (Pa) denotes the pressure of the system, *C*_CO_2_,L_^t^ (mol/m^3^) denotes the true concentration of CO_2_ in the liquid phase, and *H*_CO_2__ (Pam^3^/mol) denotes the Henry’s law constant for CO_2_ in aqueous MEA. As in the work of Akula et al.,^13^ the Henry’s law constant *H*_CO_2__ is computed using the N_2_O analogy method.^13,23,45^ In particular, for a ternary CO_2_–MEA–H_2_O mixture, the calculation involves the logarithmic mixing rule

14 $$H_{{\mathrm{CO}}_{2}}=\exp\left(w_{\mathrm{MEA}}\ln H_{{\mathrm{CO}}_{2},\mathrm{MEA}}+w_{H_{2}O}\ln H_{{\mathrm{CO}}_{2},H_{2}O}+w_{\mathrm{MEA}}w_{H_{2}O}\alpha_{\mathrm{MW}}\right)$$

where *w*_MEA_ and *w*_H_2_O_ are the mass fractions of MEA and H_2_O in the solvent, respectively; *H*_CO_2_,MEA_ and *H*_CO_2_,H_2_O_ are the Henry’s law constants of CO_2_ in pure MEA and pure H_2_O, respectively; and α_MW_ is a correction factor that accounts for nonideal mixing effects. The correction factor α_MW_, which depends on the temperature and composition of the mixture, is calculated using

15 $$\alpha_{\mathrm{MW}}=\alpha_{1}+\alpha_{2}\left(T_{L}-273.15\right)-\alpha_{3}{\left(T_{L}-273.15\right)}^{2}+\alpha_{4}w_{H_{2}O}$$

where the coefficients α_1_, α_2_, α_3_, α_4_ are considered potentially uncertain parameters, and *T*_L_ (K) denotes the temperature of the system.

Uncertainty in the chemical reaction equilibrium submodel arises from the equations relating the equilibrium constants of the aqueous-phase CO_2_–MEA–H_2_O speciation reactions to the system temperature. Due to the reactive absorption of CO_2_ in aqueous MEA solutions, the chemical equilibrium submodel must be solved simultaneously with the VLE submodel of the system. The chemistry of the aqueous phase of the CO_2_–MEA–H_2_O system is adequately represented by the reversible formation reactions (eqs 6 and 7). Although other ionic species (for example, H^+^, OH^–^, and CO_3_^2–^) appear in solution, their concentrations are shown to be negligible at the process conditions of interest.^23,44^ The full set of equations for the speciation submodel, which we use to compute *C*_CO_2_,L_^t^, and the submodel’s derivation, based on the two reversible reactions (eqs 6 and 7), are presented in detail in Akula et al.^13^ In particular, the equilibrium constants *K*_1_ and *K*_2_ of eqs 6 and 7 are related to the temperature *T*_L_ by

16 $$K_{i}=\exp\left(a_{i}+\frac{b_{i}}{T_{L}}+c_{i}\ln T_{L}\right),i=1,2$$

in which the coefficients *a*_1_, *b*_1_, *c*_1_, *a*_2_, *b*_2_, and *c*_2_ are the potentially uncertain parameters.

To obtain nominal values for the potentially uncertain Henry’s law constant coefficients α_1_, α_2_, α_3_, α_4_ and equilibrium constant coefficients *a*_1_, *b*_1_, *c*_1_, *a*_2_, *b*_2_, and *c*_2_, we invoke a parameter estimation method on empirical data from prior publications. The empirical data, previously summarized in Table 6 of Morgan et al.,^23^ report the partial pressure of CO_2_ (*P*_CO_2__) in a ternary CO_2_–MEA–H_2_O mixture at equilibrium against the mixture temperature (*T*_L_), CO_2_ loading (λ_CO_2__), and MEA mass fraction (*w*_MEA_). All data is compiled into a single data set {(*T̃*_L_^*s*^, *λ̃*_CO_2__^*s*^, *w̃*_MEA_^*s*^, *P̃*_CO_2__^*s*^)}_*s*=1_^*N*^, where (*T̃*_L_^*s*^, *λ̃*_CO_2__^*s*^, *w̃*_MEA_^*s*^, *P̃*_CO_2__^*s*^) denotes the *s* ^th^ (in a total of *N*) data point. Furthermore, the VLE and reaction equilibrium submodels enable us to predict *P*_CO2_ as an implicit function *P*_CO_2__^model^(ϑ; *T*_L_, λ_CO_2__, *w*_MEA_), in which ϑ ≔ (*a*_1_, *b*_1_, *c*_1_, *a*_2_, *b*_2_, *c*_2_, α_1_, α_2_, α_3_, α_4_) is the vector of the potentially uncertain parameters.

Nominal values and uncertainty estimates for the parameters ϑ are now obtained with the parameter estimation software tool Parmest,^59^ as follows. First, we solve the least-squares problem

17 $$\underset{\vartheta }{\min}\overset{N}{\underset{s=1}{\sum }}{\left(P_{{\mathrm{CO}}_{2}}^{\mathrm{model}}\left(\vartheta ;{\mathrm{T̃}}_{L}^{s},{\mathrm{\lambda ̃}}_{{\mathrm{CO}}_{2}}^{s},{\mathrm{w̃}}_{\mathrm{MEA}}^{s}\right)-{\mathrm{P̃}}_{{\mathrm{CO}}_{2}}^{s}\right)}^{2}$$

to obtain estimates for the entries of ϑ. As we find that the equilibrium constant parameters are highly correlated, we fix *a*_1_, *c*_1_, *a*_2_, *c*_2_ to their estimated values and resolve eq 17 accordingly to obtain updated estimates of *b*_1_, *b*_2_, α_1_, α_2_, α_3_, and α_4_. Ultimately, the uncertain parameters *q* of our optimization model (eq 1) comprise *b*_1_, *b*_2_, α_1_, α_2_, α_3_, and α_4_, and the nominal realization *q* ^nom^ is set equal to the vector (*b*_1_^*^, *b*_2_^*^, α_1_^*^, α_2_^*^, α_3_^*^, α_4_^*^) of updated estimates. The nominal values for all potentially uncertain parameters ϑ are listed in Table 2. The uncertainty in the parameters *q* = (*b*_1_, *b*_2_, α_1_, α_2_, α_3_, α_4_) is quantified by $\Sigma \in R^{6\times 6}$ , the matrix of covariances among the uncertain parameters; these covariances are calculated by Parmest along with the updated parameter estimates (see Table 3).

**Table 2:** Nominal Values of the Potentially Uncertain Thermodynamic Property Model Parameters

parameter	unit	description	nominal value
*a*_1_		HCO_3_^–^ equilibrium constant coefficient 1	3.6606 × 10^2^
*b*_1_	K	HCO_3_^–^ equilibrium constant coefficient 2	–1.3326 × 10^4^
*c*_1_		HCO_3_^–^ equilibrium constant coefficient 3	–5.5686 × 10^1^
*a*_2_		MEACOO^–^ equilibrium constant coefficient 1	1.6404 × 10^2^
*b*_2_	K	MEACOO^–^ equilibrium constant coefficient 2	–7.0701 × 10^2^
*c*_2_		MEACOO^–^ equilibrium constant coefficient 3	–2.6401 × 10^1^
α_1_		CO_2_ Henry’s law constant coefficient 1	–2.0761
α_2_	K^–1^	CO_2_ Henry’s law constant coefficient 2	3.7322 × 10^–2^
α_3_	K^–2^	CO_2_ Henry’s law constant coefficient 3	–3.2721 × 10^–4^
α_4_		CO_2_ Henry’s law constant coefficient 4	–1.1110 × 10^–1^

**Table 3:** Estimated Covariances among the Uncertain VLE and Reaction Equilibrium Property Submodel Parameters

	covariance with parameter					
parameter	*b*_1_ (K)	*b*_2_ (K)	α_1_ (−)	α_2_ (K^–1^)	α_3_ (K^–2^)	α_4_ (−)
*b*_1_ (K)	2.673 × 10^3^	1.580 × 10^3^	2.193 × 10^1^	–5.037 × 10^–1^	1.169 × 10^–3^	6.571 × 10^1^
*b*_2_ (K)	1.580 × 10^3^	1.696 × 10^3^	4.603	–2.666 × 10^–1^	1.066 × 10^–3^	3.865 × 10^1^
α_1_ (−)	2.193 × 10^1^	4.603	4.219	–7.002 × 10^–2^	2.993 × 10^–4^	3.510 × 10^–2^
α_2_ (K^–1^)	–5.037 × 10^–1^	–2.666 × 10^–1^	–7.002 × 10^–2^	1.390 × 10^–3^	–6.178 × 10^–6^	–1.720 × 10^–2^
α_3_ (K^–2^)	1.169 × 10^–3^	1.066 × 10^–3^	2.993 × 10^–4^	–6.178 × 10^–6^	2.867 × 10^–8^	5.557 × 10^–5^
α_4_ (−)	6.571 × 10^1^	3.865 × 10^1^	3.510 × 10^–2^	–1.720 × 10^–2^	5.557 × 10^–5^	2.647

To visualize how well the model represents the literature VLE data, the model predictions and the experimental data are plotted together in Figure 2. The model predictions are obtained by simultaneously solving the VLE and chemical equilibrium submodels subject to 400 off-nominal uncertain parameter realizations, sampled using the MATLAB normrnd function with mean *q* ^nom^ and standard deviations derived from the covariance matrix Σ. The model and data show good agreement, with the uncertainty in the parameters visibly impacting the model prediction at the lower temperatures of 40 and 80 °C, which are typical of absorber operation.

**Figure 2:**
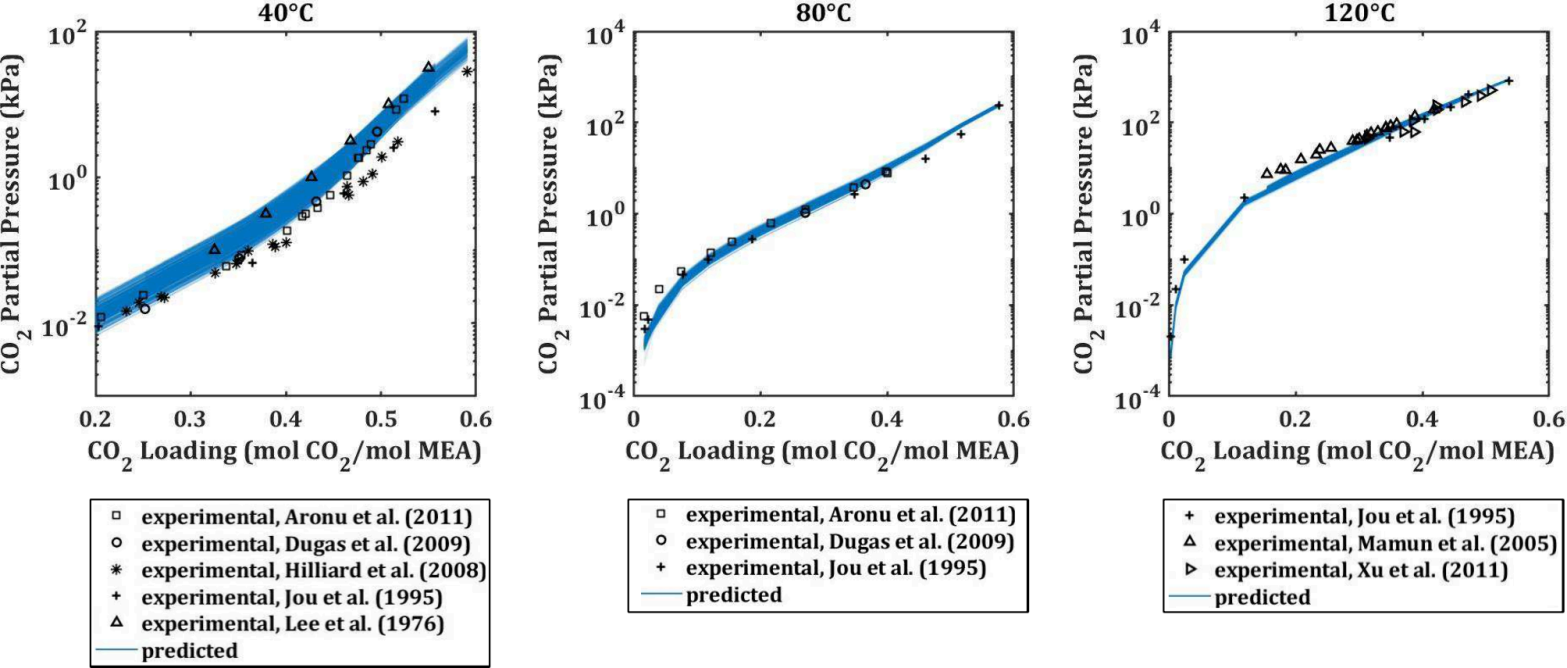
Comparison of experimental CO_2_ partial pressure data (markers) with predictions (translucent blue lines) obtained via simultaneous solution of the VLE and reaction equilibrium property submodels, subject to 400 sampled off-nominal uncertain parameter realizations, at temperatures of 40 °C, 80 °C, and 120 °C.

### 2.4 Robust Optimization Counterpart of the Deterministic Model

The deterministic model (eq 1) ignores the parametric uncertainty that was quantified in Section 2.3. Consequently, a design for the scrubbing process based on a solution of eq 1 may become operationally infeasible when the uncertain parameter *q* of the model deviates from its nominal realization *q* ^nom^. This motivates us to propose the use of robust optimization (RO) to seek economically optimized scrubbing process designs that are immunized against parametric uncertainty.

As in typical RO settings, we assume that the uncertain parameters *q* are restricted in value to a compact deterministic uncertainty set. Since the parameter estimation approach of Section 2.3 implicitly assumes that the uncertain parameters *q* follow a multivariate normal probability distribution with mean *q* ^nom^ and covariances Σ,^59^ we take the uncertainty set to be the confidence ellipsoidal region

18 $$Q^{\mathrm{conf}}\left(p\right)=\left\{q\in R^{6}|{\left(q-q^{\mathrm{nom}}\right)}^{Τ}\Sigma^{-1}\left(q-q^{\mathrm{nom}}\right)\leq \chi_{6}^{2}\left(p\right)\right\}$$

where *p* ∈ [0, 1) denotes a preselected confidence level, and χ_6_^2^(·) denotes the lower-tail quantile function of the chi-square distribution with six degrees of freedom.

For a given confidence level *p* ∈ [0, 1), the two-stage nonlinear RO counterpart of the deterministic model (eq 1) is defined by

$$\begin{array}{ccc} \underset{\left(x,z^{\mathrm{nom}},y^{\mathrm{nom}}\right)\in X^{\mathrm{nom}}}{\min}\underset{q\in Q^{\mathrm{ell}}\left(p\right)}{\max}\underset{\begin{array}{c} z\in R^{n_{z}}\\ y\in R^{n_{y}} \end{array}}{\min} & \mathrm{LCOTC}\left(x,z^{\mathrm{nom}},y^{\mathrm{nom}}\right) & \left(19a\right)\\ \mathrm{s.t.} & g\left(x,z,y\right)\leq 0 & \left(19b\right)\\ & h\left(x,z,y,q\right)=0 & \left(19c\right) \end{array}$$ 19

where the set $X^{\mathrm{nom}}$ is defined by

20 $$X^{\mathrm{nom}}=\left\{\left(x,z^{\mathrm{nom}},y^{\mathrm{nom}}\right)\in X\times R^{n_{z}+n_{y}}|\begin{array}{c} g\left(x,z^{\mathrm{nom}},y^{\mathrm{nom}}\right)\leq 0\\ h\left(x,z^{\mathrm{nom}},y^{\mathrm{nom}},q^{\mathrm{nom}}\right)=0 \end{array}\right\}$$

The objective (eq 19a) is the LCOTC evaluated subject to the nominal realization *q* ^nom^ of the uncertain parameters. We formally say that a design x∈X is *robust feasible*, or *risk-averse*, with respect to the confidence level *p*, if there exist (*z* ^nom^, *y* ^nom^) such that (*x*, *z* ^nom^, *y* ^nom^) is feasible for eq 19. Note that this occurs only if, for every $q\in Q^{\mathrm{ell}}\left(p\right)$ , there exist values $z\in R^{n_{z}}$ and $y\in R^{n_{y}}$ such that (*x*, *z*, *y*, *q*) satisfies eq 19c. Since the quantile function χ_6_^2^(*p*) of eq 18 increases monotonically as *p* increases, we have $Q^{\mathrm{ell}}\left(p\right)\subset Q^{\mathrm{ell}}\left(p^{\prime }\right)$ for any *p*′ ∈ (*p*, 1). Thus, the robust counterpart (eq 19) is relaxed [restricted], if the confidence level *p* is reduced [increased]. Further, due to eq 20, a feasible solution (*x*, *z* ^nom^, *y* ^nom^) to eq 19 is also feasible for the deterministic problem (eq 1); that is, a robust feasible design x∈X is also deterministically feasible.

To obtain a feasible solution to eq 19, we use the RO solver PyROS,^33^ which is an implementation of the cutting-set algorithm of Isenberg et al.^14^ The algorithm is an extension of that of Mutapcic and Boyd^30^ to two-stage RO problems with uncertain equality constraints. For ease of use, PyROS is designed to operate with a deterministic model and an uncertainty set as its main inputs; the robust counterpart is then automatically inferred, and a solution to the robust counterpart is automatically sought.^33^ In the algorithm of PyROS,^14,33^ the adjustability of the second-stage variables *z* to the uncertain parameters *q* is, for tractability purposes, approximated (or, more precisely, restricted) by the introduction of polynomial decision rules of a prespecified degree of 0, 1, or 2; the degree is referred to in Isenberg et al.^14^ as the decision rule order. A higher decision rule order allows for optimization over a broader range of recourse policies and thus may yield better quality solutions but may increase the time required for the algorithm to converge.^14,33^

## 3 Computational Study

### 3.1 Implementation

While the full source code used in the present work is available at https://github.com/IDAES/publications/tree/main/sherman_et_al_2026, below we discuss a few important aspects of our implementation of the model and associated optimization workflows.

#### 3.1.1 Deterministic Model

The deterministic model, eq 1 of section 2.2, was implemented using the Python-based, equation-oriented process modeling toolkit IDAES.^60^ Our implementation involved a significant effort that was aimed at enhancing the numerical robustness of the model. In particular, the IDAES diagnostic toolbox was used to guide the scaling and reformulation of the model constraints in an endeavor described in detail in Allan et al.^37^ The absorber and stripper columns were each discretized into 40 finite elements, resulting in the model size statistics shown in Table 4.

**Table 4:** Model Size Statistics for the Implemented Amine Scrubbing Process Model

symbol	description (number of)	value
*n*_*x*_	first-stage variables	12
*n*_*z*_	second-stage variables	3
*n*_*y*_	(unfixed) state variables	10113
*n*_*q*_	uncertain parameters	6
*n*_*g*_	uncertain inequality constraints	7720
*n*_*h*_	uncertain equality constraints	10113

We note that bounds were imposed on several design variables to ensure physically realistic process configurations, guided by the best engineering judgment. In particular, we required that the heights of the absorber and stripper not exceed 36 m, that their diameters not exceed 18 m, that their height-to-diameter ratios range from 1.2 to 30, and that the cross heat exchanger area not exceed 25000 m^2^. Additionally, an upper bound of approximately 80.7 MW was imposed on the maximum reboiler heat duty to ensure that the IP/LP steam extraction fraction does not exceed an upper limit of 0.85.

#### 3.1.2 Setup of the Optimization Study

With the goal of obtaining and comparing deterministically optimal (against the nominal scenario) and risk-averse model solutions for high CO_2_ capture targets, we conducted a computational study of the MEA scrubbing process model subjected to each of nine CO_2_ capture targets ranging from 90% to 99.5%. Subject to each CO_2_ capture target, we sought a nominally optimal solution and a robust feasible solution to the scrubbing process model with respect to confidence intervals of 90%, 95%, and 99%. Using an empirical postsolve approach similar to that of Isenberg et al.,^14^ the operational feasibility and quality of every solution obtained was evaluated subject to each of 400 off-nominal uncertain parameter realizations uniformly sampled from the 99% confidence interval ellipsoidal region defined by eq 18. The 400 realizations were obtained by invoking the linear transformation of eq 15 of Wang et al.^61^ on points uniformly sampled from the origin-centered unit ball through the method of Voelker et al.;^62^ thus, uniformity of the sampled realizations in the ellipsoidal region is mathematically guaranteed.^63,64^

The computational environment for the study was configured as follows. A Python 3.12.11 virtual environment was set up with installations of IDAES 2.9.0.dev0 and Pyomo 6.9.5.dev0 (along with all required dependencies). Instances of the deterministic optimization model (eq 1) were solved with GAMS 49.6.1/CONOPT 4.36.^65,66^ Solutions to instances of the RO model (eq 19) were obtained using PyROS 1.3.10,^33^ configured according to Table 5 and Appendix A. IPOPT 3.13.2,^67^ with the linear solver MA57,^68,69^ was used to initialize the model ahead of obtaining each solution and to evaluate the NGCC+CCS costing metrics for each solution found. All runs were conducted on a system of two 10-core Intel Xeon Gold 5215 CPUs, each with a clock speed of 2.50 GHz, running Ubuntu Linux 20.04 with 125 GB of RAM. Instances were solved in parallel across 10 of the 20 available CPU cores, as follows. One core was reserved for sequentially solving the deterministic model instances, while for each CO_2_ capture target of interest, one core was reserved for sequentially solving the RO counterparts defined by the target.

**Table 5:** PyROS Solver Configuration Used in the Computational Studies of the Present Work^a^

PyROS solver argument	value
local_solver	GAMS 49.6.1/CONOPT v4.36 ^64,65^ (CONOPT v3.17Q and Knitro 14.2.0 as backups^a^)
decision_rule_order	0
robust_feasibility_tolerance	10^–3^
solve_master_globally	false
bypass_global_separation	true
objective_focus	“nominal”

a The solver arguments are fully described in the official PyROS solver documentation.^70^

b PyROS affords users the option to designate backup subsolvers to be invoked whenever the main subsolver fails to return an optimal solution for a subproblem presented to it.

### 3.2 Response to CO_2_ Capture Target

Our computational study enables us to obtain deterministic and robust optimal solutions for a broad range of CO_2_ capture targets. With deterministic optimization, we successfully obtained optimal designs for all tested CO_2_ capture targets. The highest tested CO_2_ capture target for which a robust feasible design can be found depends on the confidence level of the ellipsoidal uncertainty set used. More specifically, subject to the 90% and 95% confidence levels, we obtain robust feasible designs for capture targets of up to 99.3%, whereas subject to the 99% confidence level, we obtain robust feasible designs for capture targets of up to 99.0% only. For higher CO_2_ capture targets, the PyROS solver indicates robust infeasibility, meaning that no candidate design could be identified to satisfy all constraints across the full range of uncertainties considered.

We now analyze the responses of the solutions to the CO_2_ capture target. Figure 3 shows the responses of the optimal costs and CO_2_ capture efficiency. The optimal absorber and stripper column dimensions and volumes are visualized in Figure 4, whereas Figure 5 shows the optimal heat exchanger areas. The optimal capacities of the CO_2_ capture rate, reboiler heat duty, solvent flow rates, and solvent fill are visualized in Figure 6. The second-stage decision variable values are shown in Figure 7. The states of the CO_2_-rich and CO_2_-lean solvents are visualized in Figure 8. Finally, Figure 9 summarizes the results of our empirical robust feasibility assessments. We note that, in Figures 3–8, all quantities are visualized according to their values as reported by the solvers and evaluated subject to the nominal uncertain parameter realization *q* ^nom^ (see Section 2.3).

**Figure 3:**
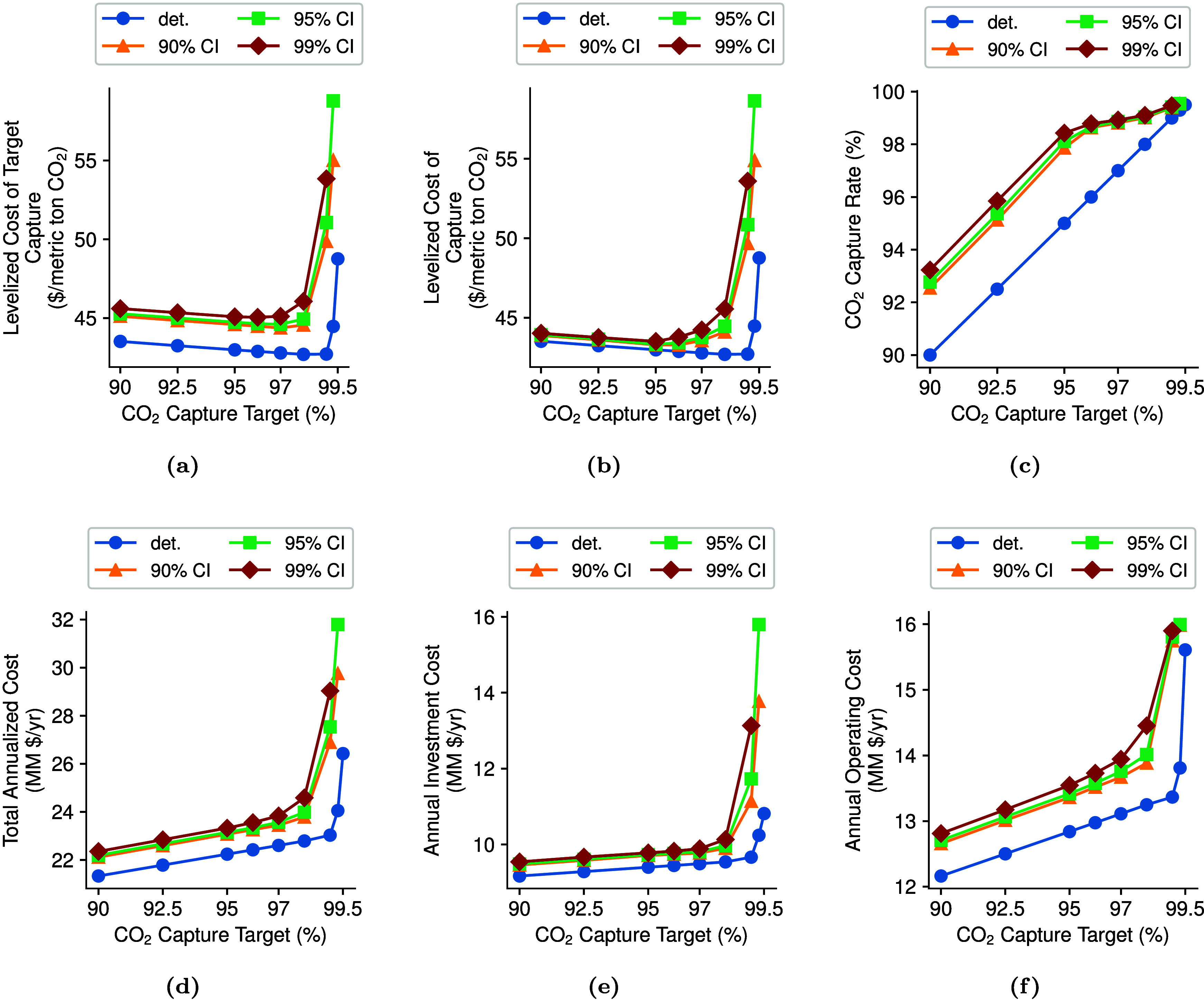
Optimal values of the (a) LCOTC, (b) LCOC, (c) (actual) CO_2_ capture rate, (d) TAC, (e) AIC, and (f) AOC, as a function of CO_2_ capture target, corresponding to the deterministically optimal (det.) and risk-averse PyROS solutions for various confidence intervals (CI).

**Figure 4:**
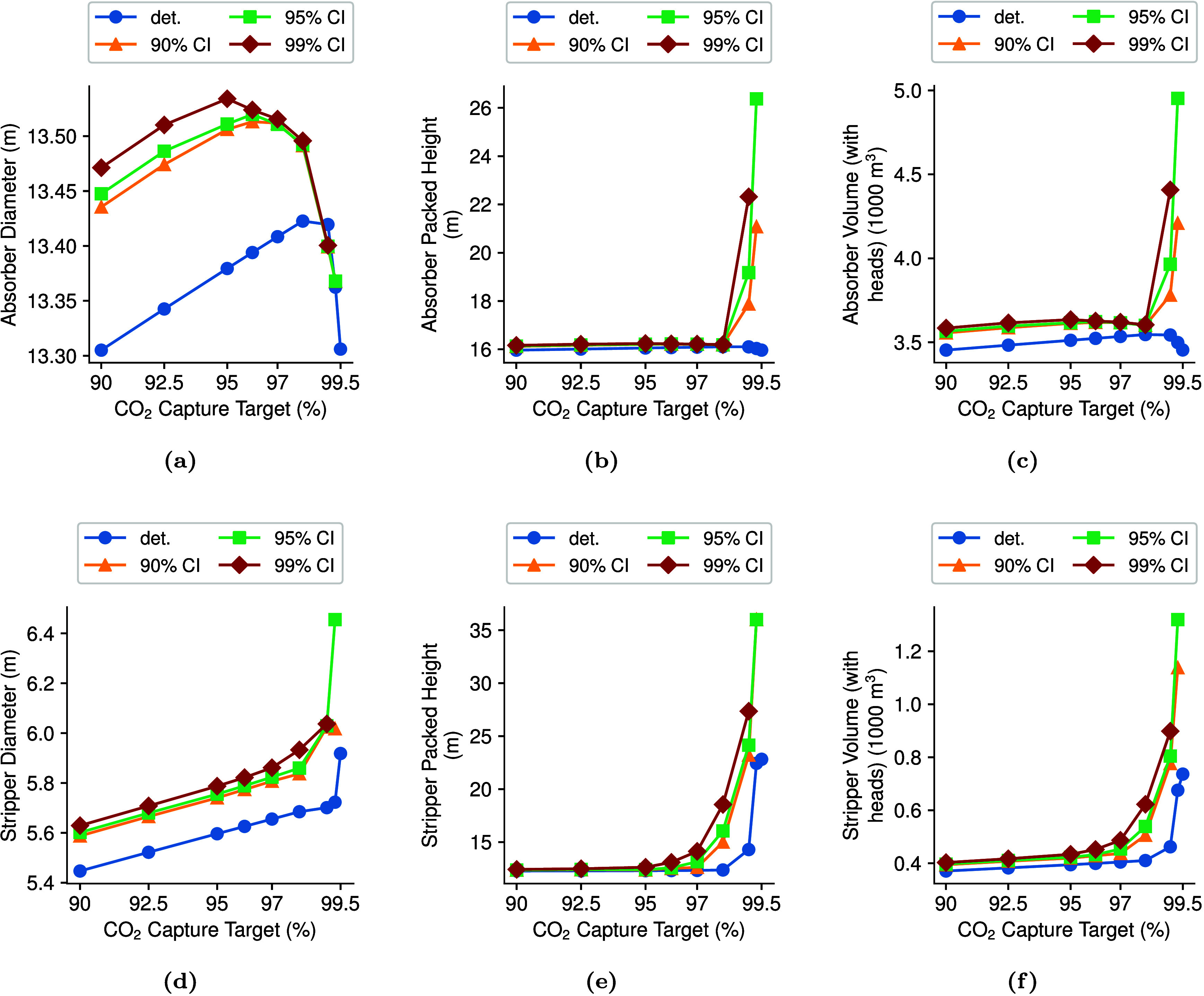
Optimal values of the (a) absorber diameter, (b) absorber packed height, (c) absorber volume, (d) stripper diameter, (e) stripper packed height, and (f) stripper volume as a function of CO_2_ capture target, corresponding to the deterministically optimal (det.) and risk-averse PyROS solutions for various confidence intervals (CI).

**Figure 5:**
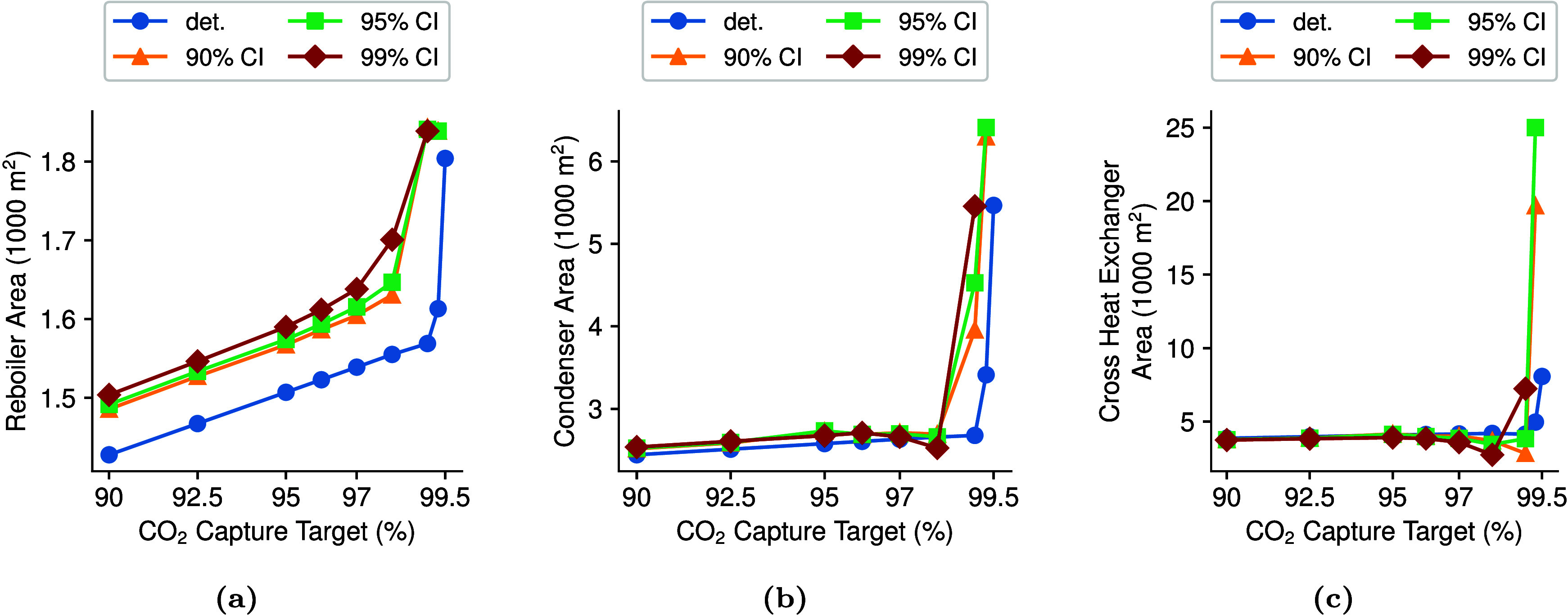
Optimal heat transfer areas of the (a) reboiler, (b) condenser, and (c) cross heat exchanger as a function of CO_2_ capture target, corresponding to the deterministically optimal (det.) and risk-averse PyROS solutions for various confidence intervals (CI).

**Figure 6:**
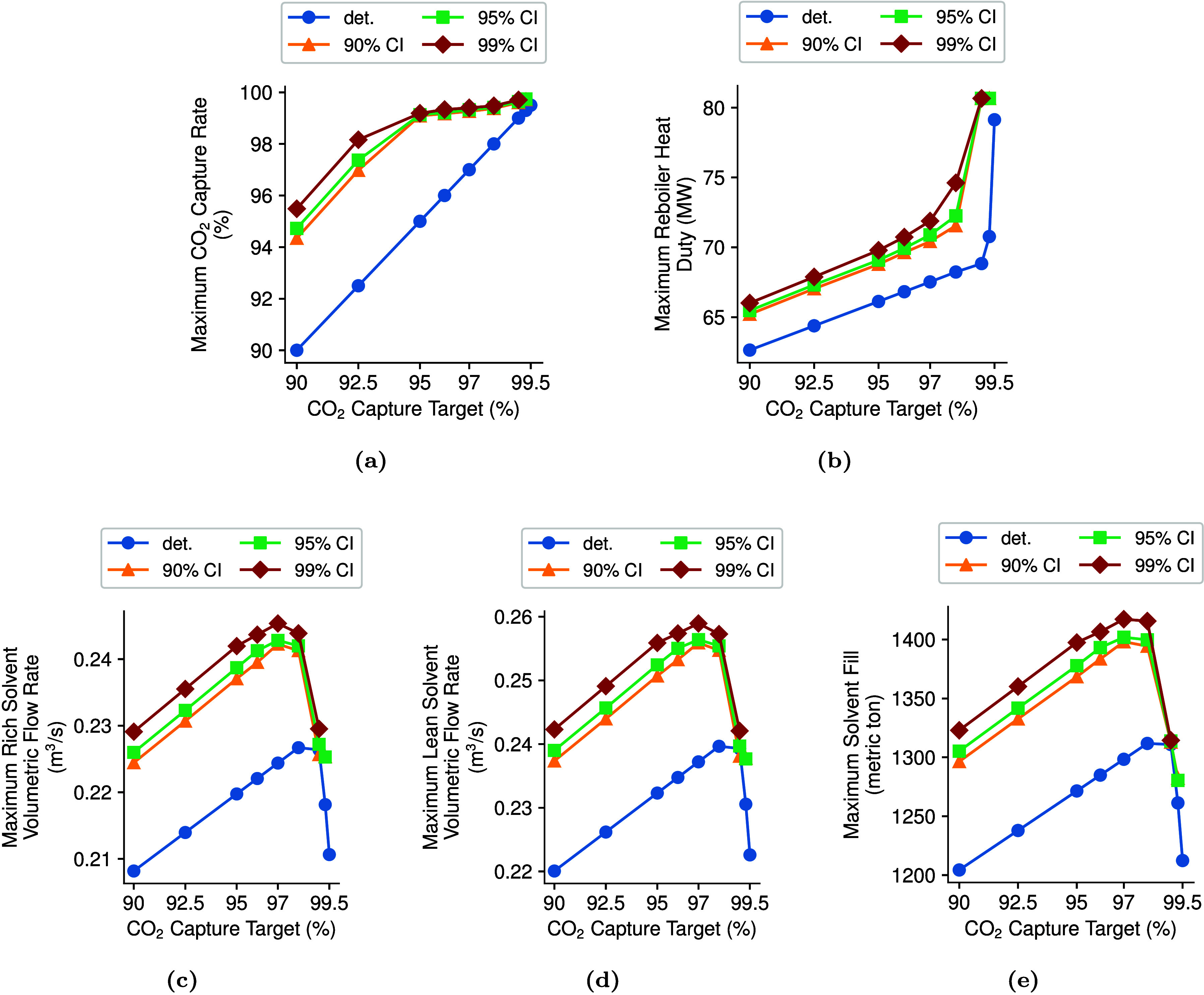
Optimal values of the maximum (a) CO_2_ capture rate, (b) reboiler duty, (c) rich solvent flow rate, (d) lean solvent flow rate, and (e) solvent fill as a function of CO_2_ capture target, corresponding to the deterministically optimal (det.) and risk-averse PyROS solutions for various confidence intervals (CI).

**Figure 7:**
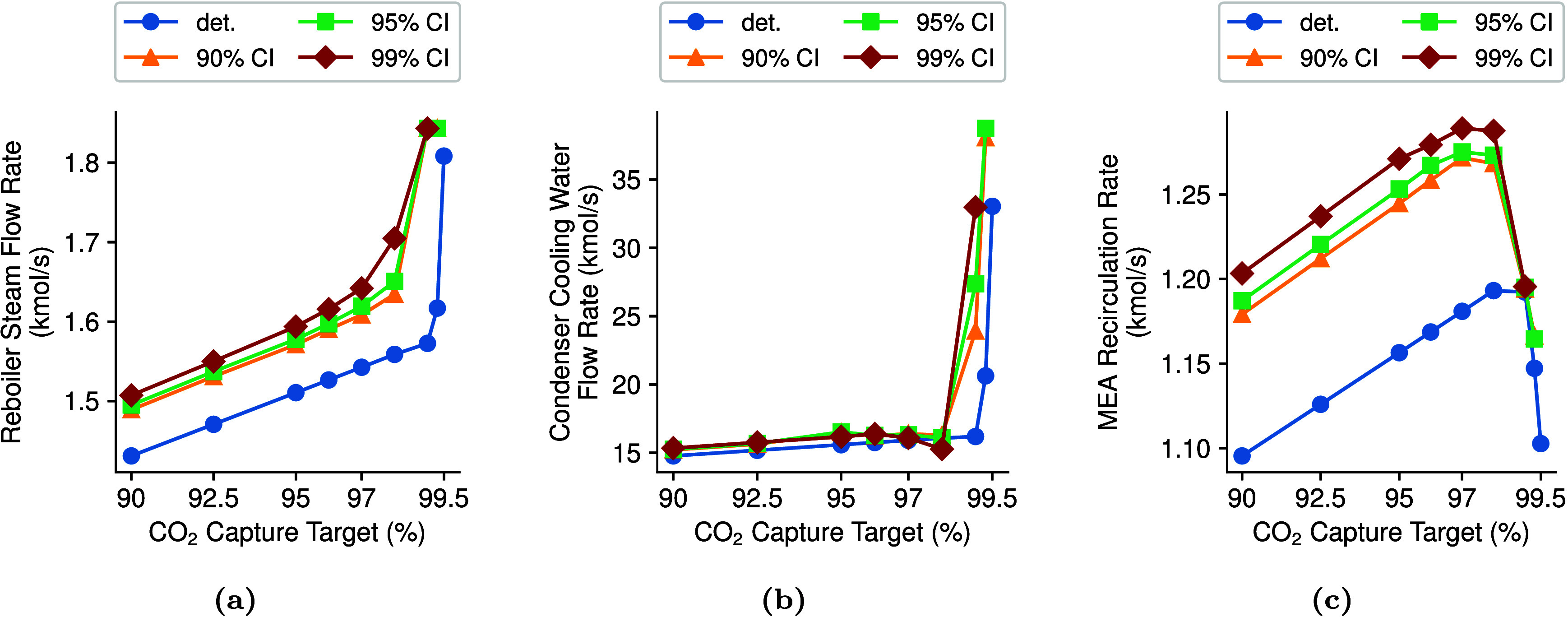
Optimal flow rates of the (a) reboiler steam utility, (b) condenser cooling water utility, and (c) recirculated MEA as a function of CO_2_ capture target, corresponding to the deterministically optimal (det.) and risk-averse PyROS solutions for various confidence intervals (CI).

**Figure 8:**
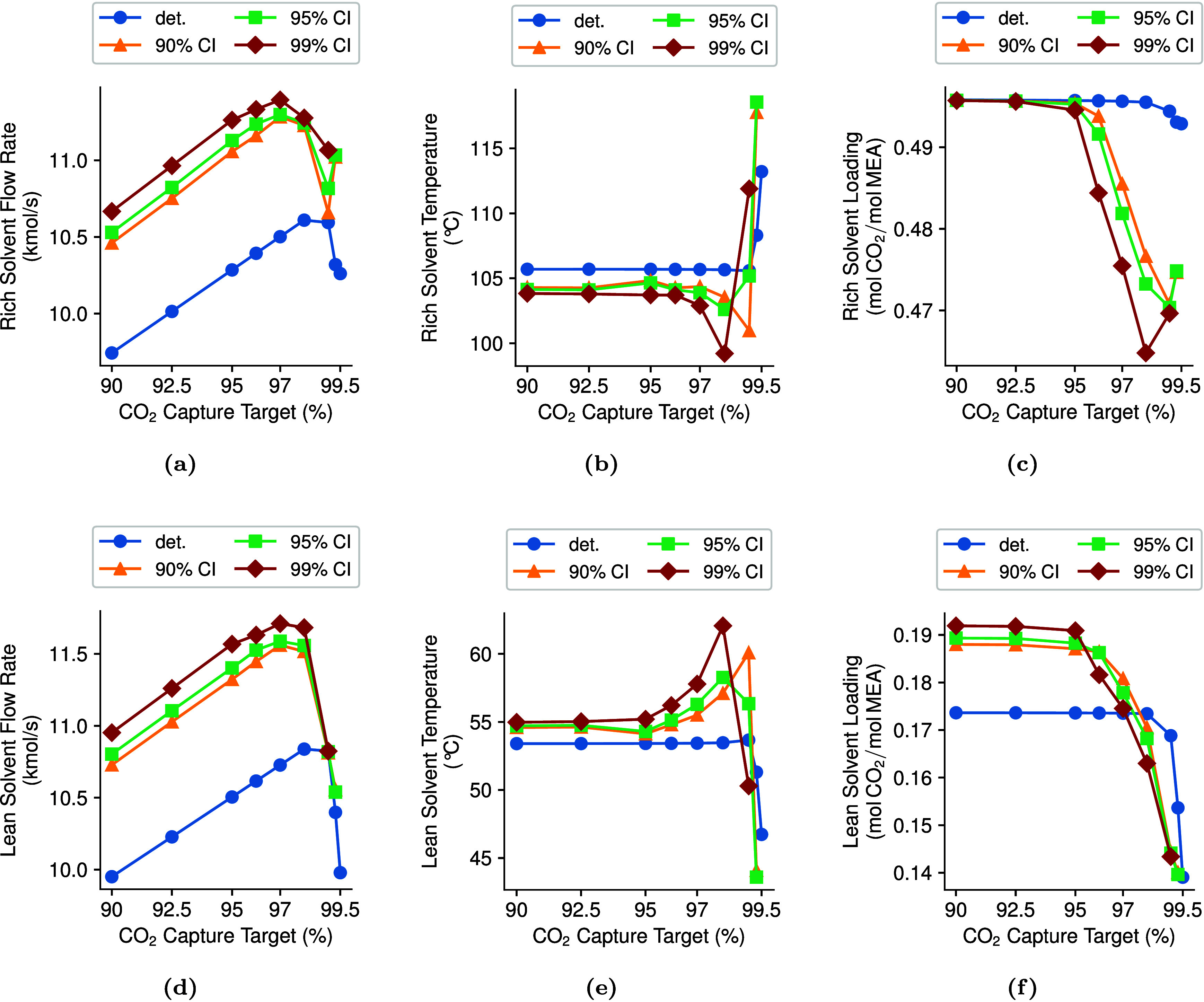
Optimal (a) stripper liquid inlet flow rate, (b) stripper liquid inlet temperature, (c) rich solvent CO_2_ loading, (d) absorber liquid inlet flow rate, (e) absorber liquid inlet temperature, and (f) lean solvent CO_2_ loading, as a function of CO_2_ capture target, corresponding to the deterministically optimal (det.) and risk-averse PyROS solutions for various confidence intervals (CI).

**Figure 9:**
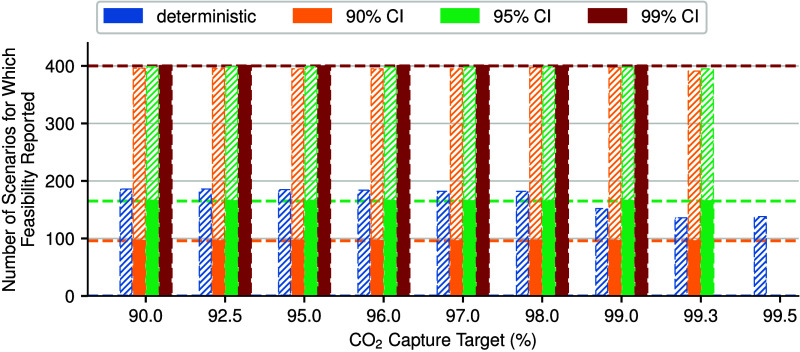
Operational feasibility statistics of the deterministically optimal and risk-averse PyROS solutions for various confidence intervals (CI). Scenarios were generated randomly according to the procedure described in Section 3.1. The hatched portion of each bar represents the out-of-sample realizations for which operational feasibility was reported. Each dashed horizontal line indicates the number of all sampled scenarios located in the ellipsoidal region of confidence level encoded in the line’s color.

As Figure 3a shows, there is a nonmonotonic relationship between the LCOTC and the CO_2_ capture target. At lower capture targets, the LCOTC decreases by around $0.10/ton CO_2_ for every 1% increment of the capture target. This reduction occurs due to eq 8 and the fact that the TAC (Figure 3d) increases more slowly than the inverse of the CO_2_ capture target flow rate. Depending on the confidence level, the LCOTC encounters a minimum at a capture target between 96% and 98%; therefore, the most economical process is designed for a capture target within that range. In contrast, at higher CO_2_ capture targets, the LCOTC increases sharply with respect to the target, and the rate of increase is more pronounced for higher confidence levels. Consequently, the LCOTC for the deterministic 99.3% capture solution is approximately 4% greater than the LCOTC for the deterministic 98.0% capture solution, whereas subject to 95% confidence ellipsoidal uncertainty, the corresponding marginal increase in the LCOTC is approximately 31%.

The differences between the optimal LCOTC values (Figure 3a) and LCOC values (Figure 3b) follow from eqs 8 and 9 and the gaps between the CO_2_ capture target and the actual CO_2_ capture rate. As Figure 3c shows, the actual CO_2_ capture rates for all robust solutions exceed their targets. This discrepancy arises from the robust feasibility requirement, which calls for ensuring that the CO_2_ capture target be met operationally for all uncertain parameter realizations within a given confidence ellipsoidal region. To achieve satisfactory capture under less favorable uncertain parameter realizations, the robustly optimized process solutions are such that the CO_2_ capture rate exceeds the stipulated target under the nominal realization. In contrast, since the deterministically optimized solutions are required to meet the CO_2_ capture target subject to only the nominal realization, there is no economic incentive or technical requirement to operate at a capture rate exceeding the stipulated target at the nominal realization. However, this jeopardizes the CO_2_ capture performance of the deterministic solutions under off-nominal realizations; the implications for the robust infeasibility of such solutions are discussed later in this section.

The cost curves of Figure 3 show that the robust feasibility requirements of eq 19 induce higher costs than the nominally optimal cost. The gap between the nominally optimal and robustly optimized LCOTC values, in particular, trends upward as the CO_2_ capture target and the confidence level of the uncertainty set are increased. At a 90% CO_2_ capture target, the LCOTC increases by up to 4% of its deterministically optimal value, whereas at a 99.3% capture target, the LCOTC increases by up to 32% of its nominally optimal value. The foregoing impact of the robust feasibility requirements on the optimized objective value has also been observed in other RO applications and is termed the *price of robustness*.^71^ Further, as discussed in Sherman et al.,^34^ the difference between the deterministically and robustly optimized objective values suggests an upper bound for the worthwhile value of performing subsequent data acquisition to reduce parametric uncertainty.

Figures 4–7 show that, for all CO_2_ capture targets, when uncertainty is accounted for, the optimal designs and operational policies are generally more conservative. As shown in Figure 4, the optimal packed heights and volumes of the absorber and stripper columns increase monotonically with the confidence level for most or all CO_2_ capture targets. Observe also that, for all CO_2_ capture targets, the reboiler area (Figure 5a), maximum CO_2_ capture rate (Figure 6a), maximum reboiler heat duty (Figure 6b), maximum solvent flow rates (Figure 6c,d), and maximum solvent fill (Figure 6e) are higher when the confidence level is increased. Similarly, the reboiler utility flow rate (Figure 7a) and MEA recirculation rate (Figure 7c) are also increased during operation. These changes are reflected in the growths of the TAC, AIC, and AOC (Figure 3d–f), as the summands of the costing expressions vary proportionately with (nonnegative powers of) many of the design and operational decision variables.

Kinetic limitations appear to dictate the optimal solution at relatively low CO_2_ capture targets. The prevailing changes to the solution, as the CO_2_ capture target is raised, are quasi-linear increases in the column dimensions (Figure 4), maximum solvent flow rates (Figure 6c–e), and MEA recirculation rate (Figure 7c). These changes are accommodated by increases in the area (Figure 5a), maximum heat duty (Figure 6b), and steam utility consumption rate (Figure 7a) of the reboiler. Notice also that for relatively low capture targets, the solvent temperatures (Figure 8b,e) and solvent CO_2_ loadings (Figure 8c,f) remain fairly constant as the capture target is changed. The range of capture targets for which these behaviors are observed is reduced as the confidence level is increased; among the deterministically optimized solutions, deviations are observed beyond 98% capture, whereas among the nonzero confidence level robust solutions, deviations are observed beyond approximately 95–96% capture.

In contrast, thermodynamic limitations appear to dictate the optimal solution at relatively high CO_2_ capture targets. Beyond a threshold CO_2_ capture target of approximately 98% if the process is optimized deterministically, or approximately 96% if the process is optimized under uncertainty, the lean solvent CO_2_ loading (Figure 8f) is reduced to establish a stronger driving force for CO_2_ removal from the flue gas in the absorption column. At the same time, the optimizer also prescribes a general increase in the stripping column dimensions (Figure 4d–f), particularly the height; the heat exchanger areas (Figure 5), particularly that of the reboiler (Figure 5a); the capacities of the CO_2_ capture rate, reboiler heat duty, and (in some cases) the solvent flows/fill (Figure 6); and the flow rates of the reboiler and condenser utility streams (Figure 7a,b). As the CO_2_ capture target exceeds 97% or 98%, the nominal flow rate of the recirculated solvent (Figure 8d), the capacities of the solvent flow rates (Figure 6c–e), and, in several cases, the dimensions of the absorption column (Figure 4c–e), particularly the diameter, can be feasibly reduced for cost savings.

The robust solutions found for ultrahigh CO_2_ capture targets (i.e., capture targets of 99% and above) are limited by the bounds on the design variables. At all robust solutions for all ultrahigh capture targets, the stripper column packed height (Figure 4e), maximum reboiler heat duty (Figure 6b), condenser area (Figure 5b), reboiler steam flow rate (Figure 7a), and condenser cooling water flow rate (Figure 7b) are increased to reduce the solvent lean loading (Figure 8f), so as to raise the solvent’s maximum CO_2_ capture rate. However, the maximum reboiler heat duty is restricted to its upper bound. Further increases to the maximum CO_2_ capture rate of the scrubbing process are achieved by raising the absorber packed height (Figure 4b) and cross heat exchanger area (Figure 5c). Collectively, these changes to the scrubbing process designs result in sharp growth of the costs (Figure 3). The ability to obtain robust feasible solutions is curtailed beyond the 99.3% capture target and 95% confidence level, for which the corresponding solution admits a cross heat exchanger area at its upper bound. Since there remains no other means to improve the maximum CO_2_ capture rate of the scrubbing process, PyROS reports robust infeasibility if the confidence level is increased or a higher capture target is specified. Hence, in Figures 3–8, no results are reported for the 99.3% capture problem subject to 99% confidence interval uncertainty or for the robust 99.5% capture problem instances.

Here, we emphasize that, as all separation subproblems were solved with only local NLP solvers, PyROS cannot inherently certify the robust feasibility of a solution. Indeed, to establish a mathematically rigorous certificate of robust feasibility, PyROS requires that the separation subproblems at the final cutting-set iteration be solved to global optimality. However, this is not possible in the present study due to the strong nonconvexities of these subproblems, making them intractable to be solved with currently available global NLP solvers. For similar reasons, approximate solution of separation subproblems has also been used in other engineering applications of cutting-set algorithms for RO.^30,72,73^

To alleviate this issue and increase our confidence in the robustness of the PyROS solutions, we subject each solution we derive in this work to the empirical robust feasibility assessment procedure described in Section 3.1.2. The results of our robust feasibility assessments, shown in Figure 9, suggest that, with full recourse, the process designs returned by PyROS are not only robust feasible, but also exhibit much stronger robustness than those found using deterministic optimization. Indeed, for every capture target and every confidence level in our study, the PyROS solution obtained subject to the uncertainty set $Q^{\mathrm{ell}}\left(p\right)$ , defined in eq 18, is operationally feasible subject to all sampled scenarios located in $Q^{\mathrm{ell}}\left(p\right)$ ; that is, all solutions may be characterized as robust feasible with respect to the uncertainty sets that were applicable in each case. Moreover, for all solutions, there are a significant number of out-of-sample scenarios for which feasibility is detected. More specifically, for all robust solutions, operational feasibility is reported subject to at least 97% of the 400 sampled scenarios. In contrast, for all deterministic solutions, operational feasibility is reported subject to fewer than half of these scenarios. Our results are examined in further detail for the 98% CO_2_ capture target solutions in Section 3.5.

### 3.3 Evaluation of Model Outputs for the Coupled NGCC+CCS System

Our implementation of the MEA scrubbing process model allows for the evaluation of its solutions via NETL’s comprehensive costing model for the NGCC+CCS system (see Section 2.2.2) to establish more rigorous cost estimates; Figures 10 and 11 visualize the results.

**Figure 10:**
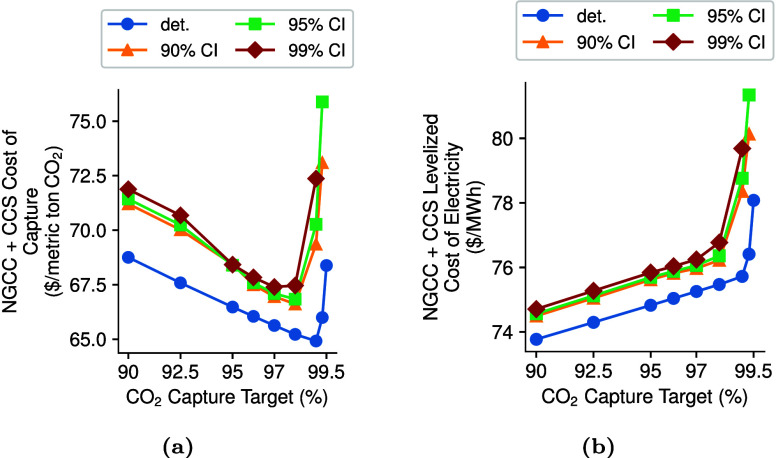
NETL costing model output values for the NGCC+CCS (a) COC and (b) LCOE as a function of CO_2_ capture target, corresponding to the deterministically optimal (det.) and risk-averse PyROS solutions for various confidence intervals (CI).

**Figure 11:**
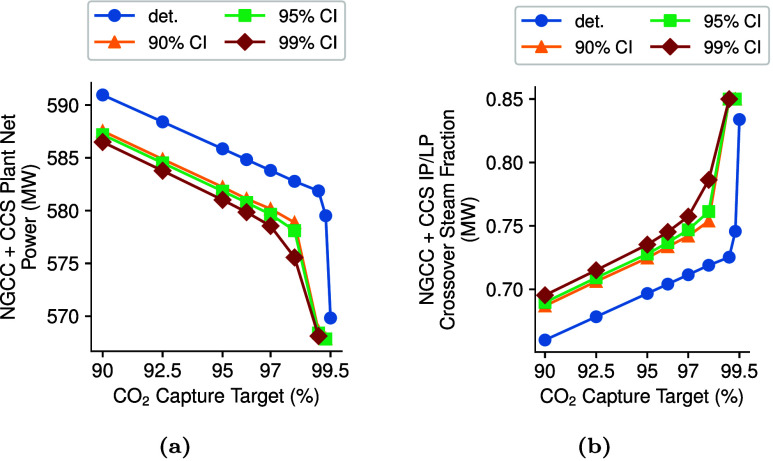
NETL’s NGCC+CCS costing model output values for the (a) NGCC plant net power production and (b) fraction of steam extracted from the NGCC power plant as a function of CO_2_ capture target, corresponding to the deterministically optimal (det.) and risk-averse PyROS solutions for various confidence intervals (CI).

The COC and LCOE of the NGCC+CCS system, as visualized in Figure 10, exhibit dissimilar trends. At CO_2_ capture targets of up to 98%, the COC (Figure 10a) decreases as the CO_2_ capture target is increased, but this effect is reversed at higher CO_2_ capture targets. In contrast, the LCOE (Figure 10a) monotonically increases with the CO_2_ capture target. The behavior of the COC at the lower capture targets follows from eq 12 and the fact that the ratio of the NGCC plant net power output (Figure 11a) to the CO_2_ capture target decreases more quickly than the LCOE grows. At CO_2_ capture targets above 98%, however, the LCOE grows more quickly than at lower capture targets; therefore, due to eq 12, the COC rises with the CO_2_ capture target.

Visualized in Figure 11, trends in the energy-related metrics of the coupled scrubbing process and the NGCC power plant reveal that the energy penalty of fitting the scrubbing process increases with the CO_2_ capture target. As Figure 11a shows, the net power production of the plant trends downward as both the CO_2_ capture target and confidence level of the uncertainty set are increased; commensurately, the fraction of steam extracted from the NGCC power plant (Figure 11b) increases. Once the CO_2_ capture target is increased to 99%, the fraction of steam extracted from the NGCC plant, which varies proportionately with the reboiler heat duty, approaches its upper bound of 85%. As discussed in section 3.2, the presence of this upper bound contributes to the robust infeasibility of the 99.3% capture instance subject to 99% confidence interval uncertainty, and of all robust optimization instances targeting 99.5% CO_2_ capture. Here, it is important to note that postulating a reduced upper bound may further limit the highest CO_2_ capture target for which feasible designs can be obtained. To counter this, one might consider introducing an external steam source, such as an auxiliary boiler, to augment the maximum quantity of steam available.

### 3.4 Computational Performance

Figure 12 summarizes the computational performance of the deterministic and PyROS solvers in the present computational study. As Figure 12a shows, solution of the deterministic optimization (i.e., 0% confidence interval) instances requires under 1 min of wall time, whereas solution of the RO instances requires approximately 15–45 min of wall time. No more than 7 PyROS iterations are required for the solution of each RO instance, as shown in Figure 12b. These performance statistics suggest that, albeit considerably higher than those of their deterministic optimization counterparts, the computational costs of obtaining robust feasible solutions or proving robust infeasibility are nonprohibitive in the process design context.

**Figure 12:**
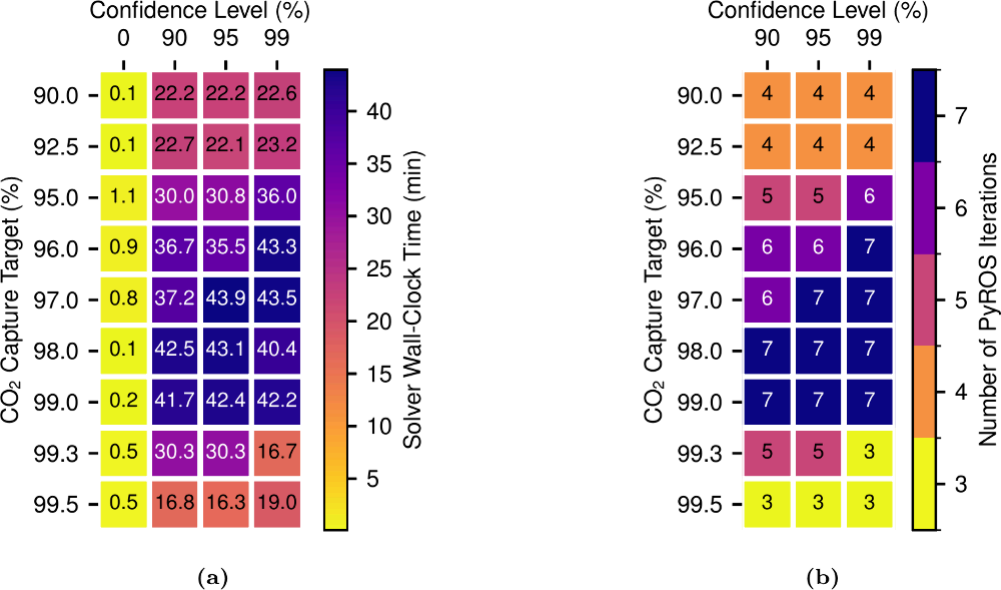
(a) Solve time and (b) PyROS iteration requirements for all process model instances addressed in the present work.

### 3.5 Detailed Comparison of Deterministic and Robust Solutions for 98% CO_2_ Capture

We now turn to a detailed comparison of the deterministically optimized and robust feasible solutions to the 98% capture design problem. Table 6 shows the comparisons of the solutions found. Our empirical evaluation of the robust feasibility and reoptimized LCOC, performed according to the method of Section 3.1.2, is visualized in Figure 13. Empirical distributions of the reoptimized costs and operational decision variables are visualized in Figures 14 and 15, respectively.

**Table 6:** Optimization Results for the 98% CO_2_ Capture Target Problem, As Reported by the Deterministic (det.) and PyROS Solvers

		value at solution for confidence interval			
quantity	unit	0% (det.)	90%	95%	99%
TAC	MM$/yr	22.79	23.78	23.98	24.59
--AIC	MM$/yr	9.54	9.90	9.97	10.13
----absorber packing cost	MM$/yr	3.78	3.84	3.84	3.84
----absorber column cost	MM$/yr	2.44	2.46	2.46	2.47
----stripper packing cost	MM$/yr	0.52	0.67	0.72	0.85
----stripper column cost	MM$/yr	0.52	0.59	0.61	0.66
----reboiler cost	MM$/yr	0.31	0.33	0.33	0.34
----condenser cost	MM$/yr	0.54	0.55	0.54	0.52
----solvent cost	MM$/yr	0.39	0.42	0.42	0.43
----other investment costs	MM$/yr	1.04	1.04	1.05	1.02
--AOC	MM$/yr	13.25	13.88	14.02	14.45
----steam utility cost	MM$/yr	12.84	13.47	13.60	14.05
----other operating costs	MM$/yr	0.41	0.41	0.41	0.40
absorber diameter	m	13.42	13.49	13.49	13.50
absorber packed height	m	16.11	16.19	16.19	16.19
stripper diameter	m	5.68	5.84	5.86	5.93
stripper packed height	m	12.37	15.00	16.06	18.55
reboiler area	1000 m^2^	1.56	1.63	1.65	1.70
condenser area	1000 m^2^	2.66	2.69	2.66	2.53
cross heat exchanger area	1000 m^2^	4.20	3.71	3.45	2.75
maximum reboiler heat duty	MW	68.23	71.54	72.25	74.62
maximum solvent fill	Gg	5.25	5.58	5.60	5.66
maximum CO_2_ capture rate	%	98.00	99.38	99.42	99.48
maximum rich solvent flow rate	m^3^/h	0.23	0.24	0.24	0.24
maximum lean solvent flow rate	m^3^/h	0.24	0.25	0.26	0.26
steam utility flow rate	kmol/s	1.56	1.63	1.65	1.70
cooling water utility flow rate	kmol/s	16.07	16.28	16.09	15.27
MEA recirculation rate	kmol/s	1.19	1.27	1.27	1.29
absorber liquid inlet flow rate	kmol/s	10.84	11.52	11.56	11.68
stripper liquid inlet flow rate	kmol/s	10.61	11.23	11.24	11.28
rich solvent CO_2_ loading	mol CO_2_/mol MEA	0.50	0.48	0.47	0.46
lean solvent CO_2_ loading	mol CO_2_/mol MEA	0.17	0.17	0.17	0.16
rich solvent temperature	°C	105.66	103.54	102.60	99.21
lean solvent temperature	°C	53.47	57.10	58.27	62.06

**Figure 13:**
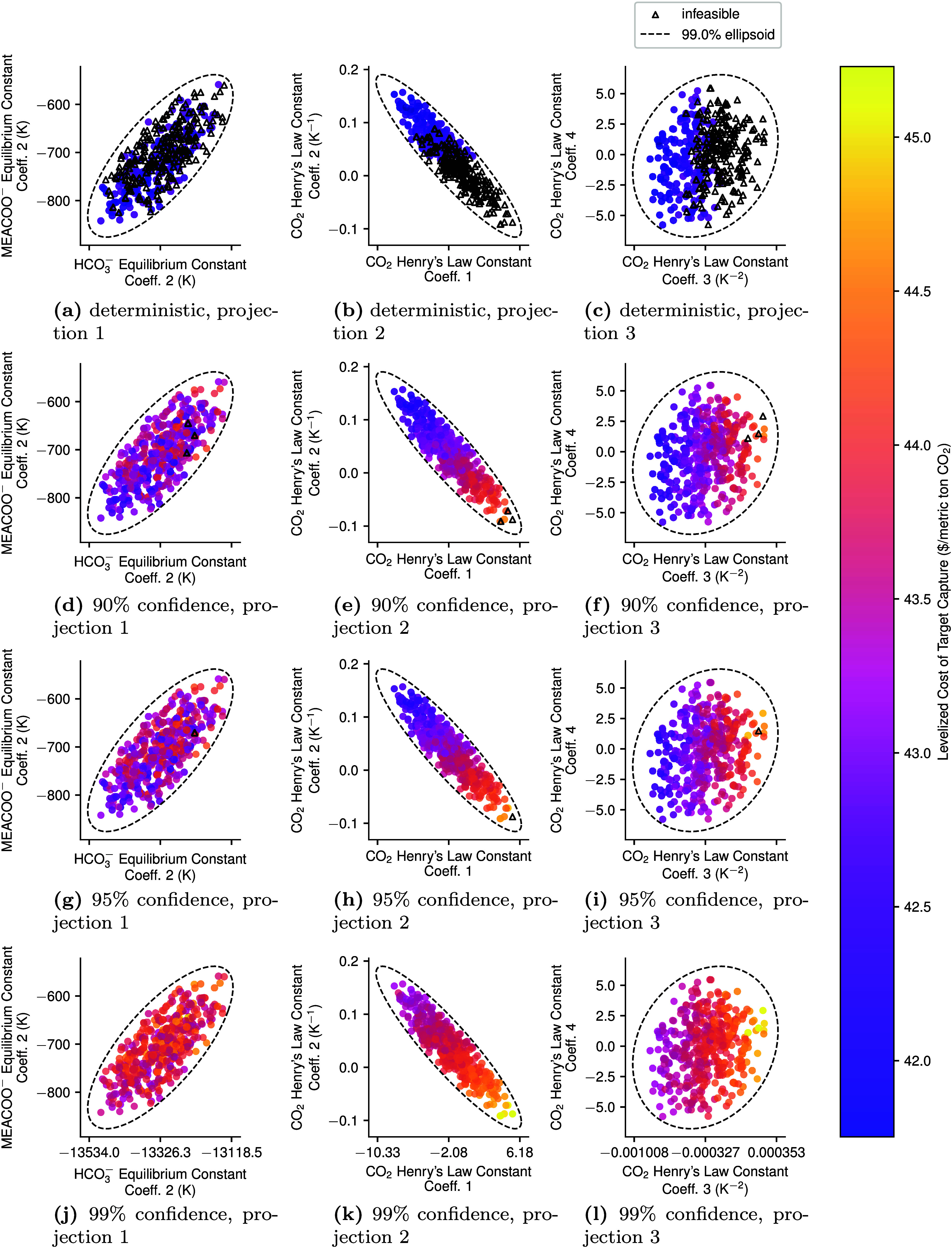
Responses of the operational feasibility and reoptimized LCOTC to the uncertain parameters for the (a)–(c) deterministic, (d)–(f) 90% confidence robust, (g)–(i) 95% confidence robust, (j)–(l) 99% confidence robust first-stage solutions to the instance featuring 98% CO_2_ capture target, evaluated for each of 400 realizations uniformly sampled from the 99% confidence ellipsoidal region. For each solution, each of the three corresponding panels depicts a unique projection of this region onto a two-dimensional, axis-oriented plane containing the center of the ellipsoid.

**Figure 14:**
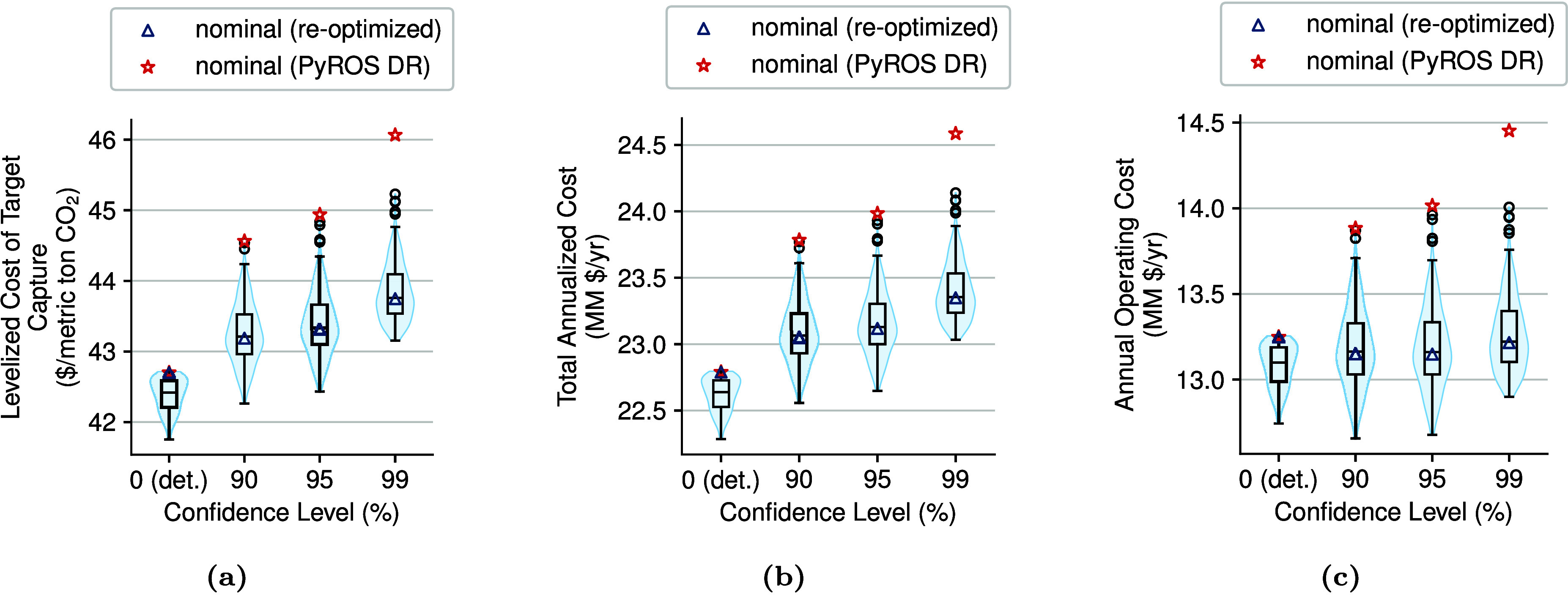
Empirical distributions of the reoptimized (a) LCOTC, (b) TAC, and (c) AOC for the instance featuring 98% CO_2_ capture target, corresponding to the deterministically optimal (det.) and risk-averse PyROS solutions for various confidence intervals (CI).

**Figure 15:**
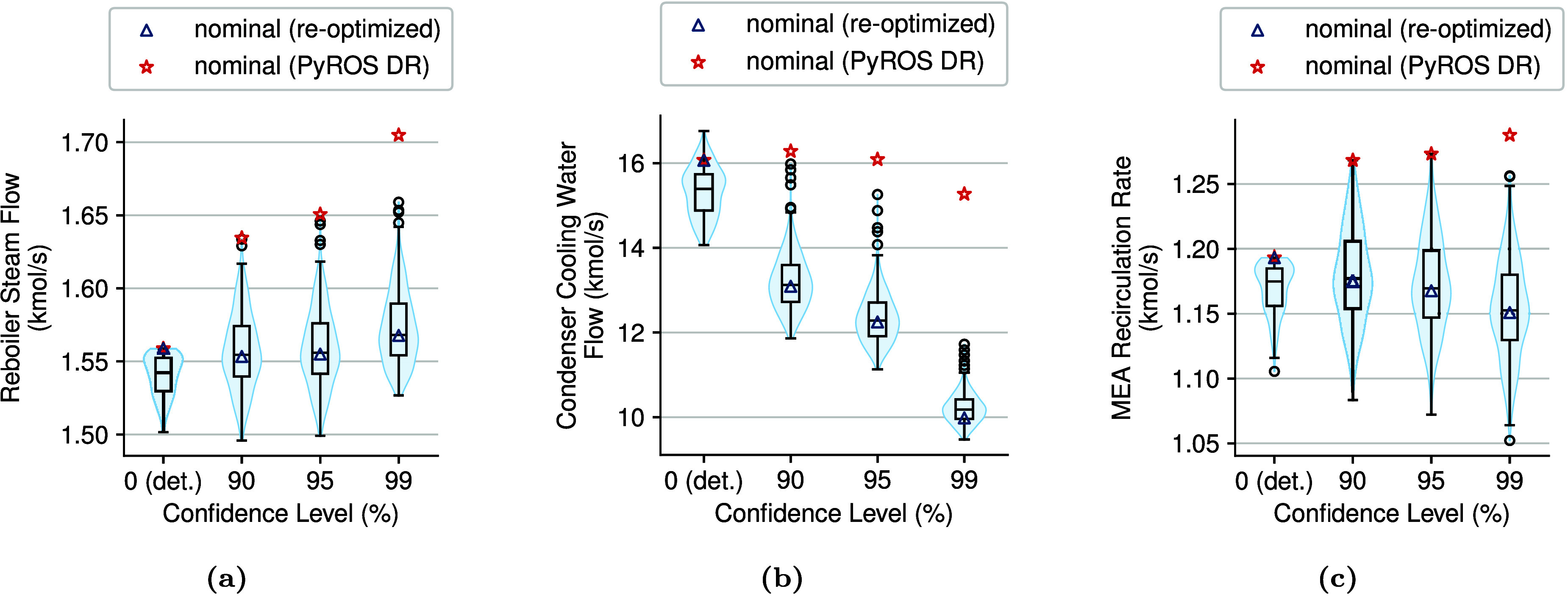
Empirical distributions of the reoptimized (a) reboiler steam utility flow rate, (b) condenser cooling water utility flow rate, and (c) MEA recirculation rate for the instance featuring 98% CO_2_ capture target, corresponding to the deterministically optimal (det.) and risk-averse PyROS solutions for various confidence intervals (CI).

In agreement with Figure 3, Table 6 shows that for a CO_2_ capture target of 98%, the robust feasible model solutions are more expensive than their deterministic counterpart. Observe that the TAC, AIC, and AOC all increase monotonically with the confidence level; in particular, the TAC of the design immunized against the 99% confidence ellipsoidal uncertainty set is approximately 8% higher than that of the nominally optimal design. The most significant changes to the AIC contributors are increases in the stripper column, stripper packing, and absorber packing costs and reductions in the stripper condenser cost. Moreover, an increase in the reboiler steam utility cost accounts for much of the growth of the AOC.

The cost differences among the nominally feasible and robust feasible process designs are reflected in discrepancies among the values of the design and operating variables. As Table 6 shows, the column dimensions of the robust feasible designs are greater than those of their nominally feasible counterparts; the most notable change is an increase in the height of the stripper column. We observe that the reboiler area increases monotonically as the uncertainty set is enlarged, but a reduced heat transfer area is required for the cross heat exchanger and, for the 99% confidence interval, the condenser. There are also significantly greater requirements for the maximum solvent flow rates, reboiler duty, and MEA (solvent) fill. We also see that the changes in the AOC, which consists mostly of the reboiler steam utility expenditure, closely follow changes to the reboiler steam utility flow rate.

For the case of 98% capture, the changes in the optimized process designs and operational policies with respect to the ellipsoidal confidence level appear to be driven by thermodynamic limitations. To achieve robust feasibility at higher confidence levels, the process must be designed such that it can operate at CO_2_ capture rates exceeding the target under more favorable uncertain parameter realizations, so the target can be met under less favorable ones. Consider, for example, the nominal realization, subject to which the optimal solvent flow rates, CO_2_ loadings, and temperatures are shown in Table 6. Clearly, the solvent flow rates of the robust feasible solutions are higher, and the lean solvent loadings are marginally lower, to allow for a higher CO_2_ removal rate in the absorption column. These changes are afforded by the increases in the column dimensions, MEA recirculation, heat exchanger utility flow rates, and maximum reboiler heat duty specifications. The solvent-temperature requirements are not as stringent. For a more operationally reliable process design, sufficient CO_2_ removal in the stripping column affords a higher lean solvent temperature and lower rich solvent temperature, so the cross heat exchanger area requirements are reduced.

Motivated by the discussion of Section 3.2, our empirical assessments of the 98% CO_2_ capture target solutions show that the robust feasible solutions are significantly more operable, albeit consistently more expensive, than their deterministic counterpart. As Figure 13a–c shows, in agreement with Figure 9, the nominally optimized design is operationally feasible for fewer than 50% of the sampled scenarios. In contrast, as Figure 13d–l shows, the robustly optimized designs are operationally feasible for over 99% of the sampled scenarios. Notably, each PyROS-optimized solution is confirmed to be feasible subject to all sampled realizations located within its corresponding confidence ellipsoid. Notice that, statistically, the reoptimized LCOTC grows in value as the confidence level is increased.

The scatter plots of Figure 13 show that, for all solutions, the reoptimized LCOTC is more sensitive to the uncertain Henry’s law constant parameters than it is to the uncertain speciation reaction equilibrium constant parameters. The responses to the latter, which are shown in Figure 13a,d,g,j, exhibit no clear trend in the reoptimized cost. In contrast, the responses to the Henry’s law constant parameters, shown in Figure 13b,c, e,f, h,i, and k,l, show visible patterns of the LCOTC being influenced by these parameters. The stronger response to the Henry’s law constant parameters is likely attributable to their more direct impact on the VLE within the reboiler and condenser, as the VLE ultimately influences the steam and cooling water utility requirements, and thus, the operating costs.

The empirical distributions of the costs, shown in Figure 14, suggest that for the robustly optimized designs, a fully flexible recourse policy results in strictly lower (operating) costs than those incurred by using the static policy prescribed by PyROS. As seen in Figure 14, the costs evaluated subject to the static recourse policy exceed not only the reoptimized nominal costs but also the reoptimized worst-case costs (i.e., the maxima of the cost distributions). Moreover, the gaps among the nominal reoptimized costs across confidence levels are much lower than the gaps among the costs evaluated subject to the static recourse policies. In particular, the nominal price of robustness (in terms of the LCOTC) of the 99% confidence interval solution is reduced to 1% after reoptimization subject to a full recourse policy, down from 8% when it is subject to the static recourse policy.

Reductions in the costs after reoptimization subject to a fully flexible recourse policy follow from reductions in the recourse decision variable values, with which the operating costs vary proportionately. Since under a fully flexible recourse policy the recourse decision variables are not restricted by any explicit decision rules, they can be wholly determined by the optimizer and be highly adjustable to each of the tested uncertain parameter realizations, as necessary to minimize the operating costs in each case. As Figure 15 shows, for all risk-averse solutions, the values of all three second-stage variables after reoptimization with fully flexible recourse are reduced by a margin of up to 35% across all of the tested parameter realizations. Due to these reductions, the reoptimized nominal and median values of these variables are usually lower, and always no more than 1% higher, than their counterparts at the deterministically optimal solution. The impacts of reoptimizing the recourse variables on the costs are noticeable. Observe that the distribution of the AOC (Figure 14c) closely follows that of the reboiler steam utility flow rate (Figure 15a), as the reboiler steam utility cost generally comprises more than 95% of the AOC. The observed cost reductions suggest that reoptimizing the operation of the system subject to a fully flexible recourse policy provides a more reliable economic assessment of the robust feasible solutions obtained in the present study.

## 4 Conclusions

In this work, we presented an application of the two-stage RO solver PyROS to a detailed model for the design and operation of a conventional amine scrubbing process for postcombustion CO_2_ capture, subject to capture targets ranging from 90% to 99.5% and to uncertainty in the VLE and chemical reaction equilibrium property submodel parameters. Restricted to optimization over the space of static operational recourse policies, PyROS is able to provide risk-averse solutions for CO_2_ capture targets of up to 99.3%. For CO_2_ capture targets of up to 98%, the levelized cost of target capture (LCOTC) of the robustly optimized process is nominally only 8% higher than that of the deterministically optimized process, whereas for higher CO_2_ capture targets, the marginal increase in the LCOTC is as much as 32%. As we have shown, these cost increases can be significantly reduced by reoptimizing the operation of the process, subject to a fully flexible recourse policy. Our work extends the body of literature on the application of nonlinear RO methodologies, particularly the cutting-set algorithm of PyROS, to risk-averse process design.

## Appendix A: Choice of PyROS Separation Priorities for Robust Solution of MEA Scrubbing Process Model

As previously shown in Table 4 (Section 3.1), our MEA scrubbing process model has a sizable number of uncertain inequality constraints (that is, *n*_*g*_ > 7000). By default, PyROS (sequentially) solves a separation problem for each of the *n*_*g*_ scalar inequality constraints in each iteration, in accordance with Section 2.2 of Isenberg et al.^14^ However, since the solution of each separation problem requires approximately 10 s of wall time in our implementation, attempting to obtain a robust solution to any instance with the default configuration to perform our computational study is impractical. We therefore opted to prioritize approximately 30 of the inequality constraints, specified in Table [Table tblA1], and skip the solution of the separation problems for the remaining constraints. As Figure 9 (Section 3.2) shows, the results of our postsolve empirical assessments of the robust feasibility of all model solutions obtained with PyROS suggest that skipping most of the separation problems does not cause PyROS to return solutions that are robust infeasible subject to full recourse adaptivity.

**A1 tblA1:** Uncertain Inequality Constraints of the MEA Scrubbing Process Model Corresponding to the PyROS Separation Problems That Were Prioritized[Table-fn tblA1-fn1]

Pyomo component name in model implementation	description of inequality constraint(s)	number of constraints
fs.CO2_lower_bound	CO_2_ capture target constraint	1
fs.design_epigraph_cons	epigraph constraints for the operational capacities of the reboiler duty, solvent flow rates, and CO_2_ capture mass flow rate	4
fs.absorber_section.absorber.UB_flood_ratio[0.0,x]	upper bound constraint for absorber column flooding fraction at normalized height *x* = 1/40, 2/40, ..., 21/40	21
fs.stripper_section.stripper.UB_flood_ratio[0.0,0.025]	upper bound constraint for stripper column flooding fraction at normalized height 0.025	1
fs.stripper_section.stripper.LB_flood_ratio[0.0,1.0]	lower bound constraint for stripper column flooding fraction at normalized height 1	1
fs.stripper_section.reboiler.condensate_temperature_outlet	bound constraints for the reboiler utility outlet temperature	2
fs.stripper_section.condenser.DeltaT_cold_end[0.0]	lower bound constraint for the condenser cold end temperature delta	1
fs.stripper_section.condenser.DeltaT_hot_end[0.0]	lower bound constraint for the condenser hot end temperature delta	1
fs.max_cond_temp_out	upper bound constraint for the condenser process-side vapor outlet stream temperature	1

a Separation problems were skipped for all uncertain inequality constraints in the model that are not listed here.
